# Potential transceptor AtNRT1.13 modulates shoot architecture and flowering time in a nitrate-dependent manner

**DOI:** 10.1093/plcell/koab051

**Published:** 2021-02-12

**Authors:** Hui-Yu Chen, Shan-Hua Lin, Ling-Hsin Cheng, Jeng-Jong Wu, Yi-Chen Lin, Yi-Fang Tsay

**Affiliations:** Institute of Molecular Biology, Academia Sinica, Taipei 11529, Taiwan

## Abstract

Compared with root development regulated by external nutrients, less is known about how internal nutrients are monitored to control plasticity of shoot development. In this study, we characterize an *Arabidopsis thaliana* transceptor, NRT1.13 (NPF4.4), of the *NRT1/PTR/NPF* family. Different from most NRT1 transporters, NRT1.13 does not have the conserved proline residue between transmembrane domains 10 and 11; an essential residue for nitrate transport activity in CHL1/NRT1.1/NPF6.3. As expected, when expressed in oocytes, NRT1.13 showed no nitrate transport activity. However, when Ser 487 at the corresponding position was converted back to proline, NRT1.13 S487P regained nitrate uptake activity, suggesting that wild-type NRT1.13 cannot transport nitrate but can bind it. Subcellular localization and β-glucuronidase reporter analyses indicated that NRT1.13 is a plasma membrane protein expressed at the parenchyma cells next to xylem in the petioles and the stem nodes. When plants were grown with a normal concentration of nitrate, *nrt1.13* showed no severe growth phenotype. However, when grown under low-nitrate conditions, *nrt1.13* showed delayed flowering, increased node number, retarded branch outgrowth, and reduced lateral nitrate allocation to nodes. Our results suggest that NRT1.13 is required for low-nitrate acclimation and that internal nitrate is monitored near the xylem by NRT1.13 to regulate shoot architecture and flowering time.

## Teaser

Nitrate transporter/transceptor NRT1.13 monitors xylem 12 nitrate level to regulate shoot architecture and flowering time.

## Introduction

For plants to survive, developmental plasticity, for example root architecture, shoot architecture, and floral transition, is sophisticatedly modulated by the integration of internal and external signals (Nicotra et al., 2010; de Jong and Leyser, 2012). For example, temperature and photoperiod are two well-known external signals that both regulate floral transition (Song et al., 2013). In the temperature pathway, reduced expression of the negative regulator *FLOWERING LOCUS C* (*FLC*) is a key process in promoting flowering after a period of low temperature in *Arabidopsis thaliana*. However, expression of *FLC* can also be repressed by internal signals of the autonomous pathway. Apart from light and temperature, nutrients such as nitrate act as external signals that affect floral transition (Castro Marin et al., 2011; Kant et al., 2011; Liu et al., 2013; Yuan et al., 2016; Gras et al., 2018). More key players in the nutrient signaling pathway need to be further characterized to understand how nutrient status is sensed and integrated with other pathways.

Nitrate is one of the primary nitrogen sources for plants. Two types of transporters in the NRT1 (NPF) and NRT2 families are involved in nitrate acquisition (Wang et al., 2012; Nacry et al., 2013; Krapp et al., 2014). After nitrate is acquired from soil, the xylem is the major route for long-distance nitrate transport from root to above-ground tissues. Root-to-shoot nitrate allocation is regulated positively by NRT1.5 (NPF7.3) and negatively by NRT1.8 (NPF7.2) and NRT1.9 (NPF2.9), which are expressed in different types of cells. Regulation of root-to-shoot nitrate allocation is important for stress acclimation (Lin et al., 2008; Li et al., 2010; Wang and Tsay, 2011; Chen et al., 2012). In terms of shoot nitrate allocation, NRT1.7 (NPF2.13), NRT1.11 (NPF1.2), and NRT1.12 (NPF1.1) can remobilize nitrate from old or mature leaves, via the phloem, to satisfy the high nutrient demand of young leaves (Fan et al., 2009; Hsu and Tsay, 2013). *NRT1.6/NPF2.12*, *NRT2.7*, and *NPF5.5*, expressed in embryos or siliques, are important for nitrogen or nitrate content of seeds (Chopin et al., 2007; Almagro et al., 2008; Leran et al., 2015). In addition, the CHLORIDE CHANNEL (CLC) and SLOW ANION CHANNEL-ASSOCIATED HOMOLOGUES (SLAC and SLAH) are involved in vacuolar nitrate storage or nitrate-mediated regulation of stomatal closure (De Angeli et al., 2006; Geiger et al., 2011). Through these transporters and channels, nitrate is properly allocated into different tissues and organelles for efficient nitrate utilization.

In addition to being a nutrient source, nitrate acts as a molecular signal-regulating gene expression and plant development (Crawford, 1995; Vidal et al., 2015). Referred to as the “primary nitrate response,” expression of several nitrate-related genes is induced within 10 min (and reaches a maximum within 30 min) after nitrate exposure (Hu et al., 2009). CHL1 (NRT1.1/NPF6.3)—a dual-affinity nitrate transporter involved in nitrate uptake—also functions as a transceptor to monitor changes in the external nitrate concentration and attenuate the primary nitrate response, a rapid nitrate-induced transcriptional response, according to its phosphorylation status (Ho et al., 2009). In response to low-nitrate, protein kinase CIPK23 phosphorylates CHL1 at the T101 residue, and phosphorylated CHL1 leads to a low-level primary nitrate response. Several transcription factors in this pathway have been identified (Vidal et al., 2015). For example, NLP7 can bind directly to the promoters of these nitrate-related genes, and nuclear accumulation of NLP7 is regulated by nitrate (Marchive et al., 2013). Thus, through the cooperation of the transceptor, kinases, and transcription factors, the primary nitrate response can prime the plant to assimilate nitrate when it becomes available.

To ensure nitrate is efficiently utilized for plant growth, nitrate signaling is also integrated with other signals to regulate plant development. For example, to optimize nitrate acquisition, nitrate affects primary root growth, lateral root density, and lateral root elongation in distinct ways (Forde, 2014). In *Arabidopsis*, the transceptor CHL1 (NRT1.1/NPF6.3) is involved in the interplay between nitrate and auxin to repress lateral root growth under low-nitrate conditions (Krouk et al., 2010). In *Medicago truncatula*, the transporter MtNPF6.8 (which is in the same family as CHL1) is involved in the interplay between nitrate and abscisic acid (ABA) in regulating primary root growth (Pellizzaro et al., 2014). Compared with root architecture, less is known about if and how the plasticity of shoot development is regulated by nitrate. In this study, characterization of *Arabidopsis thaliana* NRT1.13 provides insights into how nitrate is sensed in shoots to regulate flowering time, shoot architecture, and lateral nitrate allocation.

## Results

### S487P mutation can restore the nitrate transport activity of NRT1.13

**Figure 1:**
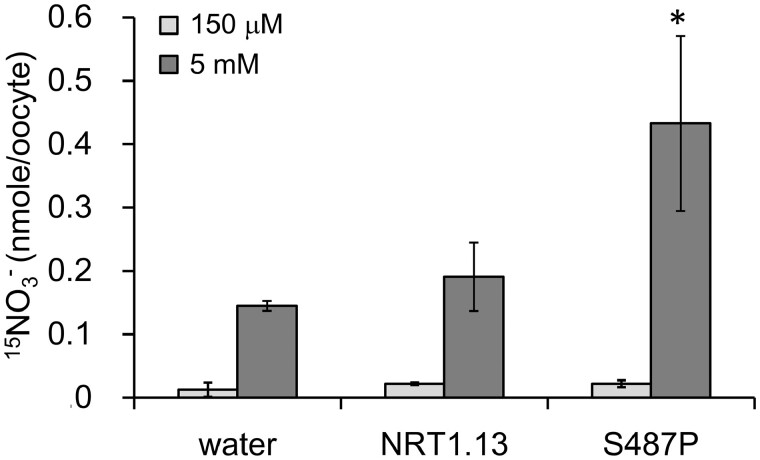
NRT1.13 shows no nitrate uptake activity, but S487P conversion restores its nitrate transport activity. High- and low-affinity nitrate transport activities of injected oocytes were assessed by incubating with 150 µM and 5 mM K^15^NO_3_ buffer, respectively, at pH 5.5 for 2.5 h and the ^15^${\text{NO}}_{3}^{–}$ contents were determined as described in the Methods. Values are the mean ± SD of five or six oocytes. Similar results were obtained from three independent frogs. (*, *P* < 0.05, Student’s *t*-test, compared with water-injected oocytes; Supplemental Data set 1).

NRT1.13 (NPF4.4), a member of the *NRT1/PTR* transporter family, shares 37% sequence identity with CHL1 (Tsay et al., 2007). CHL1 is a dual-affinity nitrate transporter involved in uptake that also functions as a nitrate transceptor to trigger the nitrate-induced transcriptional response (Liu et al., 1999; Ho et al., 2009). Our previous study showed that the Pro492 residue, in the cytosolic loop between the 10th and 11th transmembrane domains, is important for the nitrate transport activity of CHL1, but is not required for its nitrate-sensing function (Ho et al., 2009). This proline residue is highly conserved in the NRT1/PTR family. Out of 53 NRT1 (PTR) transporters in *Arabidopsis*, only three members—NRT1.13 (NPF4.4), NRT1.14 (NPF4.3), and NPF2.2—do not have the proline residue in the corresponding position (Supplemental Figure 1). In NRT1.13, the corresponding residue at position 487 is serine. As expected, *Xenopus* oocytes expressing *Arabidopsis* NRT1.13 showed little or no nitrate transport activity under either high- or low-nitrate conditions (Figure 1). When Ser487 was replaced by proline, the mutated NRT1.13 (S487P), with similar expression levels (Supplemental Figure 2), showed enhanced nitrate uptake activity under high-nitrate conditions (Figure 1). This result confirms that the conserved proline residue at that position is important for the transport activity of NRT1/PTR transporters. Since S487 is not in the substrate binding pocket, restoration of the nitrate transport activity of the NRT1.13 (S487P) mutant suggests that despite being incompetent for nitrate transport, the wild-type form of NRT1.13 is able to bind nitrate. To test whether wild-type NRT1.13 can bind nitrate, we performed a microscale thermophoresis binding assay using purified NRT1.13 protein. As shown in Supplemental Figure 3, the resulting binding isotherms clearly demonstrate that NRT1.13 can bind nitrate.

### NRT1.13, localized in plasma membrane, is expressed in xylem parenchyma cells

**Figure 2:**
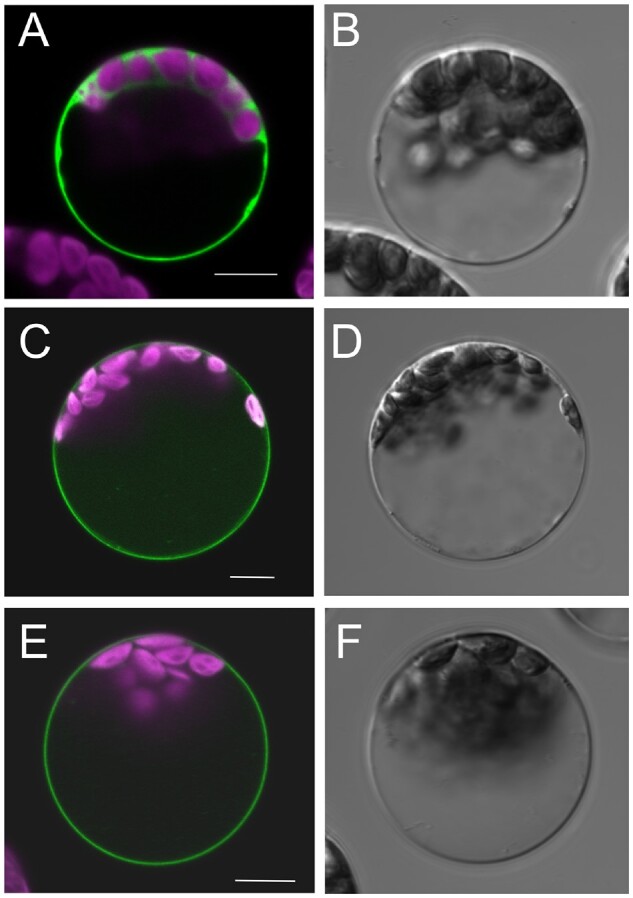
NRT1.13 is localized in the plasma membrane. (A) (C) (E) are the overlap images of the GFP (green) and chlorophyll (magenta) fluorescence. (B) (D) (E) are the bright-field images. GFP alone (A, B), NRT1.13-GFP (C, D) and NRT1.13-S487P-GFP (E, F) was transiently expressed in *Arabidopsis* mesophyll protoplasts and scanned by confocal laser microscopy. Bars = 20 µm. Similar patterns were observed in at least 30 protoplasts in three batches of independent experiments.

**Figure 3:**
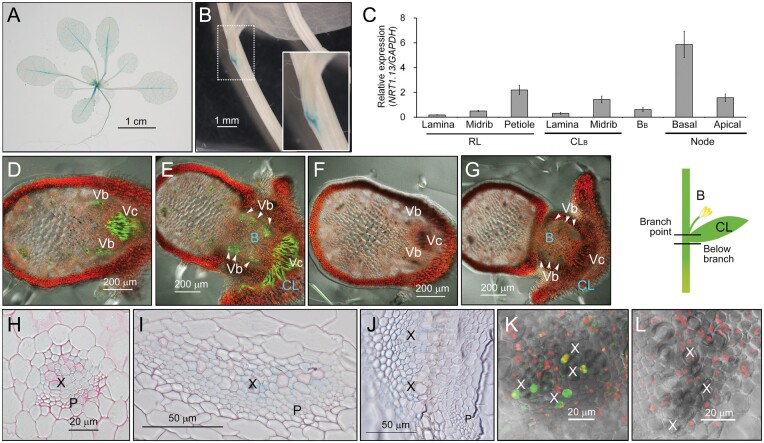
*NRT1.13* is expressed in xylem parenchyma cells with higher levels at branch points. (A) and (B) *P_NRT1.13_:GUS* expression pattern in a 24-day-old plant at the vegetative stage (A) or in the branch point of the inflorescence stem in a 44-day-old plant at the reproductive stage (B). The inset is an enlargement of the branch point. (C) RT-qPCR analysis of *NRT1.13* expression. Total RNA was isolated from the vegetative and inflorescence tissues of 25- and 33-day-old Col-0 plants, respectively. Values are means ± SE of six independent plants. (RL, rosette leaves; CL_B_, basal cauline leaf; B_B_, basal branch). (D) to (G) Cross-sectional images below branch points (D, F) and at the branch point (E, G) of *P_NRT1.13_:NRT1.13-GFP* (D, E) and control plants (F, G) grown with 2 mM KNO_3_ for 21∼25 days. (H) to (J) Enlarged vascular images from sections of petiole (H), mid-rib of cauline leaf (I), and node (J) from 24-day-old (H) or 59-day-old (I, J) *P_NRT1.13_:GUS* plants. A similar pattern was observed in another independent line. (K) and (L) Enlarged vascular images of nodes from *P_NRT1.13_:NRT1.13-GFP* (K) and control plants (L) grown with 0.2 mM KNO_3_ for 54 days. (CL, cauline leaf; B, branch; P, phloem; X, xylem; Vc, vascular tissues connecting to cauline leaf; Vb, vascular tissues connecting to branch).

Subcellular localization of NRT1.13 was analyzed by transiently expressing NRT1.13:GFP in *Arabidopsis* mesophyll protoplasts. As shown in Figure 2, the fluorescence signal is external to the chloroplast signal, indicating that NRT1.13 is localized in the plasma membrane. The tissue-specific expression pattern of *NRT1.13* was determined by histochemical assay of *P_NRT1.13_:GUS* transgenic plants. At the vegetative stage, the *NRT1.13* promoter was highly active in the major veins of rosette leaves (Figure 3A). After bolting, in addition to the major veins of rosette leaves, the *NRT1.13* promoter drove expression at the major veins of cauline leaves and the node of inflorescence stems (Figure 3B). Expression of *NRT1.13* in major veins and nodes was further validated by reverse transcription-quantitative PCR (RT-qPCR; Figure 3C). Interestingly, the basal node showed higher *NRT1.13* expression than the apical node.

To further characterize *NRT1.13* spatial expression at the nodes, cross sections of inflorescence stems in *P_NRT1.13_:NRT1.13-GFP* transgenic and control plants were examined (Figure 3D – G). As shown in the cross section of the node directly below the branch point in Figure 3D, GFP signals were already detectable in the enlarged vascular bundle (labeled as Vc) that would subsequently be linked to the cauline leaf and the interconnected vascular bundle (labeled as Vb) that would subsequently be linked to the branch. At the branch point of the node, where the cauline leaf and branch are expanded, signals of NRT1.13-GFP were detected at the vasculature of the cauline leaf and branch (Figure 3E). In control plants, there was no GFP signal at the corresponding position of the node (Figure 3F, G). These data suggest that NRT1.13 was mainly expressed in the converged vascular bundles at the node.

To further reveal in which cells in the vascular tissue *NRT1.13* is expressed, cross sections of *P_NRT1.13_:GUS and P_NRT1.13_:NRT1.13-GFP* were examined under higher magnification. GUS activities were observed around the xylem, whether in the petiole of the rosette leaf (Figure 3H), the major vein of the cauline leaf (Figure 3I), or at the node (Figure 3J). Consistent with these results, GFP signals were also detected in cells next to the xylem in *P_NRT1.13_:NRT1.13-GFP* (Figure 3K), but no signals were found in control plants (Figure 3L). These data indicate that *NRT1.13* was expressed in the xylem parenchyma cells.

### The late-flowering phenotype of *nrt1.13* is nitrate-dependent and *FLC*-dependent

**Figure 4:**
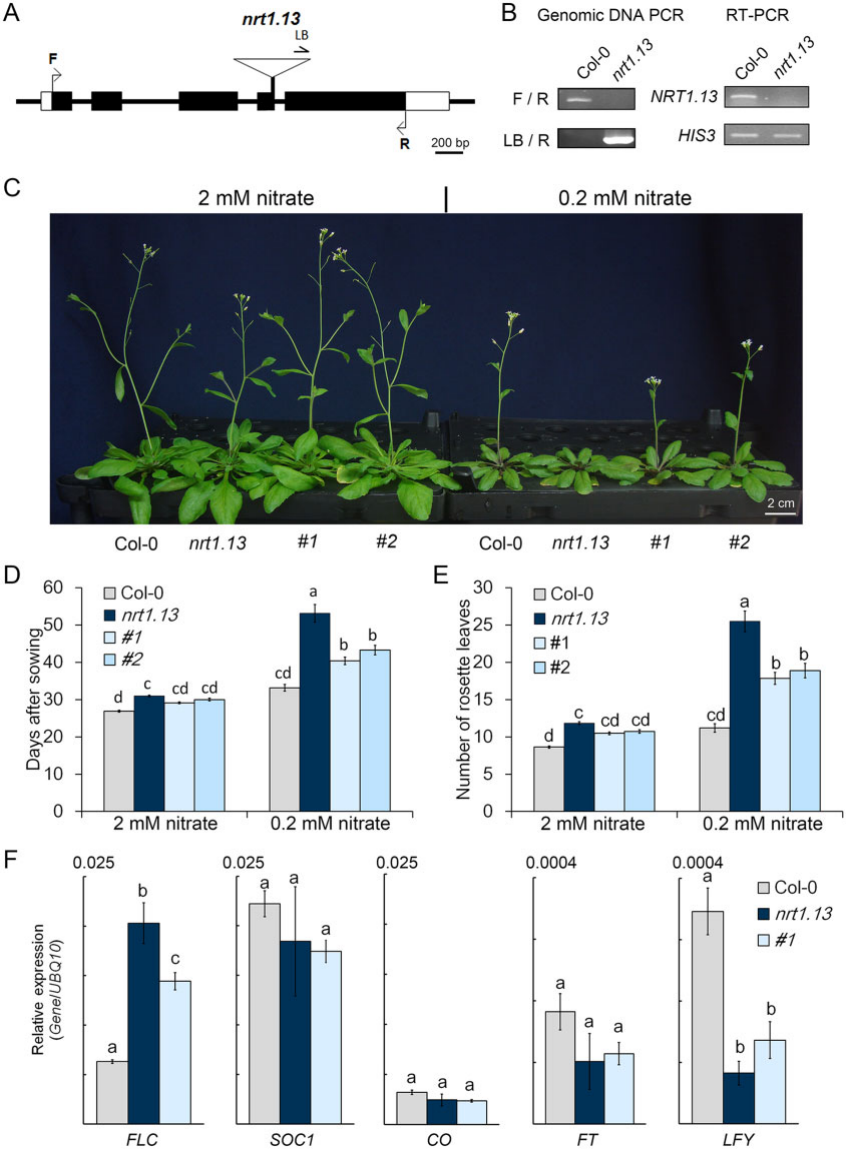
The late-flowering phenotype of *nrt1.13* is more severe under low-nitrate conditions. (A) Schematic of the T-DNA insertion site of the *nrt1.13* mutant. In *nrt1.13*, the T-DNA was inserted into the fourth exon. Black boxes, coding region; white boxes, untranslated region; F and R, the forward and reverse primer, respectively, used for genomic DNA PCR and RT-PCR; LB, left border primer of T-DNA. (B) Genomic DNA PCR and RT-PCR analyses of *nrt1.13*. Total RNA was obtained from petioles of indicated plants. *HIS3*, the internal control of RT-PCR. (C) Photo of 43-day-old plants grown hydroponically with 2 or 0.2 mM nitrate. *P_NRT1.13_:NRT1.13-GFP/nrt1.13 #1* and *#2* are two independent complementation lines. (D) and (E) Flowering time of plants grown under normal (2 mM) and low (0.2 mM) nitrate, indicated as days after sowing (D) or measured as the number of rosette leaves at bolting (E). Values are means ± SE of 25 independent plants. Statistical analysis comprised one-way ANOVA with a Tukey *post hoc* test (*P*<0.05; Supplemental Data set 1). Similar results were obtained in four independent experiments. (F) Expressions of *FLC*, *CO*, *FT*, *SOC1*, and *LFY* measured by RT-qPCR. Total RNA was isolated from whole leaves of 25-day-old plants supplied with 0.2 mM nitrate. Values are the means ± SE of three independent samples. Statistical analysis comprised one-way ANOVA with a Tukey HSD *post hoc* test (*P* < 0.05). Similar results were obtained from two additional experiments.

To assess the physiological role of NRT1.13 in plants, *nrt1.13*—a T-DNA-inserted mutant showing no NRT1.13 transcript (Figure 4A, B)—was obtained from SAIL (Syngenta Arabidopsis Insertion Library) (Sessions et al., 2002). When plants were supplied with a normal concentration of nitrate (2 mM), the flowering time of *nrt1.13* was delayed for 3 to 4 days compared with the wild-type Col-0 (Figure 4C, D). The late-flowering phenotype of *nrt1.13* could be partially rescued by introducing *NRT1.13-GFP* driven by a 2Kb *NRT1.13* promoter in the two independent complementation lines, *P_NRT1.13_:NRT1.13-GFP/nrt1.13 #1* and *#2* (Figure 4D, E). To assess whether the late-flowering phenotype of *nrt1.13* is nitrate-dependent, flowering time and leaf numbers of plants grown under normal-nitrate (2 mM) and low-nitrate (0.2 mM) conditions were compared. In Col-0, flowering time as well as leaf number at bolting showed a slight difference between normal and low-nitrate conditions, with flowering delayed for ∼4 days and leaf numbers increased by ∼3 under low-nitrate conditions (Figure 4D, E). However, in *nrt1.13*, flowering was delayed dramatically, by approximately 20 days, and leaf number at bolting increased by 14 under the low-nitrate condition (Figure 4D, E). Under low-nitrate conditions, the delayed flowering of *nrt1.13* was partially recovered in complementation lines *#1* and *#2* (Figure 4D, E). To further test the role of NRT1.13 in flowering regulation, we isolated another NRT1.13 mutant, *nrt1.13-2*, which also displayed a late-flowering phenotype similar to that of *nrt1.13* (Supplemental Figure 4). Taken together, these data show that *NRT1.13* affects the floral transition in a nitrate-dependent manner, and that the major difference between the wild-type and mutant lines in terms of flowering time mainly arises under low-nitrate conditions.

To elucidate how *NRT1.13* affects flowering time, plants were grown with 2 mM or 0.2 mM KNO_3_ for 16 or 25 days, and the shoots were collected for RNAseq analysis. As listed in Table 1, 23 genes (including *NRT1.13*) showed a > 1.8-fold change between wild type and mutant under low-nitrate conditions. One of these 23 genes is *FLOWERING LOCUS C* (*FLC*), a repressor and integrator in the vernalization and autonomous pathways. Expression of *FLC* was significantly higher in the *nrt1.13* mutant, particularly under low-nitrate conditions. Nevertheless, as shown in Supplemental Table 1, expression of genes involved in the vernalization and autonomous pathway, as well as others linked to the photoperiod, gibberellin, temperature, and aging pathways, was not changed in the *nrt1.13* mutant, suggesting that NRT1.13 regulates *FLC* expression and flowering time independently of these known pathways.

**Table 1:** Genes with alternated expression in *nrt1.13* under 0.2 mM KNO_3_, nitrate-limited condition in RNA-seq analysis

	Days after sawing	16 DAS				25 DAS			
	Nitrate (mM)	0.2		2		0.2		2	
**Locus**	**Description**	**FC**	***P***	**FC**	***P***	**FC**	***P***	**FC**	***P***
**absolute FC ≥1.8 under nitrate-limited condition at 16 and 25 DAG**									
AT5G10140	FLC	**1.97**	0.00	1.40	0.00	**2.75**	0.00	1.78	0.00
AT3G22235	CYSTM8	**1.88**	0.00	1.76	0.00	**3.17**	0.00	**2.11**	0.00
AT3G08690	UBC11	**–1.98**	0.00	–1.78	0.00	**–1.90**	0.00	–1.79	0.00
AT1G33440	NRT1.13	**–2.53**	0.00	**–2.19**	0.00	**–2.39**	0.00	**–2.02**	0.00
**absolute FC ≥1.8 under nitrate-limited condition at 16 DAG**									
AT2G30766	unknown protein	**1.97**	0.04	**2.37**	0.00	1.03	0.85	1.41	0.00
AT5G50665	unknown protein	**1.81**	0.00	–1.14	0.21	–1.22	0.17	1.11	0.43
AT4G08870	ARGAH2	**–1.83**	0.00	–1.37	0.00	1.13	0.29	–1.63	0.00
AT2G18280	TLP2	**–1.83**	0.00	–1.61	0.00	–1.61	0.00	–1.60	0.00
AT4G15210	BAM5	**–1.92**	0.00	–1.09	0.64	1.48	0.12	**–2.79**	0.00
ATCG00020	PSBA	**–2.03**	0.00	–1.05	0.80	1.06	0.79	**5.53**	0.00
**absolute FC ≥1.8 under nitrate-limited condition at 25 DAG**									
AT4G22485	LTP/protease inhibitor	–1.35	0.03	1.24	0.08	**2.40**	0.00	1.74	0.00
AT2G14560	LURP1	1.23	0.04	1.12	0.02	**1.95**	0.00	1.15	0.00
AT1G14200	RING/U-box superfamily protein	1.64	0.00	1.12	0.16	**1.91**	0.00	1.27	0.00
AT5G03350	Legume lectin family protein	–1.02	0.83	–1.01	0.79	**1.89**	0.00	1.01	0.84
AT5G01600	FER1	1.22	0.55	–1.75	0.00	**1.89**	0.02	**–5.32**	0.00
AT2G40750	WRKY54	1.04	0.72	1.07	0.34	**1.88**	0.00	1.22	0.00
AT5G60900	RLK1	1.02	0.83	1.10	0.19	**1.85**	0.00	1.10	0.07
AT1G35710	LRR protein kinase	–1.13	0.27	1.08	0.30	**1.83**	0.00	1.03	0.50
AT3G48280	CYP71A25	1.76	0.00	1.74	0.00	**1.83**	0.00	1.78	0.00
AT5G24420	PGL5	1.19	0.02	1.36	0.00	**1.81**	0.00	–1.03	0.83
AT2G23130	AGP17	1.04	0.60	1.06	0.32	**–1.83**	0.00	–1.42	0.00
AT4G08950	EXORDIUM	–1.01	0.89	–1.02	0.77	**–1.86**	0.00	–1.64	0.00
AT5G45430	Protein kinase	1.18	0.64	–1.73	0.05	**–2.96**	0.01	–1.21	0.63

FC: fold-change of gene differential expression comparing *nrt1.13* with wild type, positive value means up-regulation and negative value means down-regulation in the *nrt1.13*; FC > 1.8 or FC< 1.8 are underlined; P: *P*-value.

Expression of several key floral integrators was further confirmed by RT-qPCR. As shown in Figure 4F and consistent with our RNAseq data, expression of *FLC* was increased in *nrt1.13* compared with Col-0 at 25 DAG under low-nitrate conditions, but neither *CONSTANS* (*CO*, an activator in the photoperiod pathway) nor *SUPPRESSOR OF OVEREXPRESION OF CONSTANS 1* (*SOC1*) exhibited any difference. *FLOWERING LOCUS T* (*FT*) and *LEAFY* (*LFY*) represent convergence points for various signals, but their expression levels were too low to allow further RNAseq analysis here. When examined by RT-qPCR, expression of *FT* was not altered under our experimental conditions, whereas expression of *LFY* was significantly decreased in the *nrt1.13* mutant (Figure 4F). Transceptor CHL1 is involved in the primary nitrate response (Ho et al., 2009). As shown in Supplemental Figure 5, the primary nitrate response in roots and shoots of *nrt1.13*—as assessed using *NRT2.1* and *NIA2* as marker genes, respectively—was similar to that of wild type, indicating that NRT1.13 is not involved in the primary nitrate response. Taken together, these data indicate that *NRT1.13* controls the floral transition by a pathway that regulates the expression of *FLC* and *LFY*.

**Figure 5:**
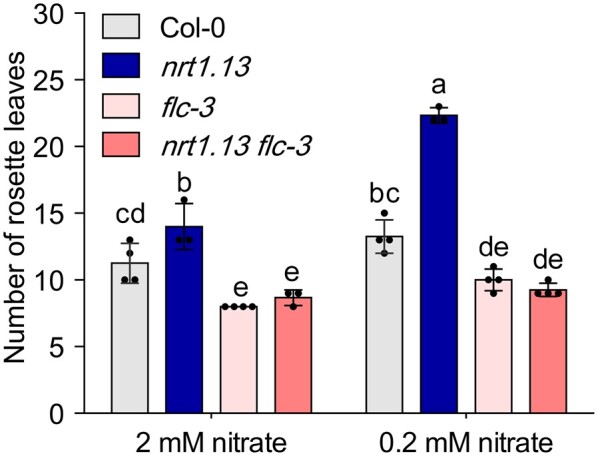
FLC is involved in the late-flowering phenotype of the *nrt1.13* mutant. The flowering time of plants grown under normal (2 mM) or low (0.2 mM) nitrate, measured as the number of rosette leaves at bolting. Values represent means ± SD of 3∼4 independent plants. Statistical analysis by one-way ANOVA with Tukey HSD *post hoc* test (p < 0.05; Supplemental Data set 1). Similar results were obtained from four experiments.

*LFY* is known to act downstream of *FLC* (Srikanth and Schmid, 2011; Song et al., 2013), so *FLC* could be the critical player in the *NRT1.13*-controlled pathway. To test whether FLC plays a major role in the nitrate- and NRT1.13-regulated pathways, we measured and compared flowering times of single and double mutants. As shown in Figure 5, under both our normal- and low-nitrate conditions, the *flc* mutant flowered earlier than the wild type. More interestingly, the nitrate-dependent late-flowering phenotype of the *nrt1.13* mutant was not manifested in the *flc nrt1.13* double mutant, indicating that *FLC* is required for the late-flowering phenotype observed in the *nrt1.13* mutant. Flowering times of the *flc nrt1.13* double mutant were similar to those of the *flc* mutant, consistent with our expression analysis, so we conclude that *FLC* functions downstream of *NRT1.13* to control nitrate-dependent flowering.

### *NRT1.13* regulates lateral nitrate allocation at nodes

**Figure 6:**
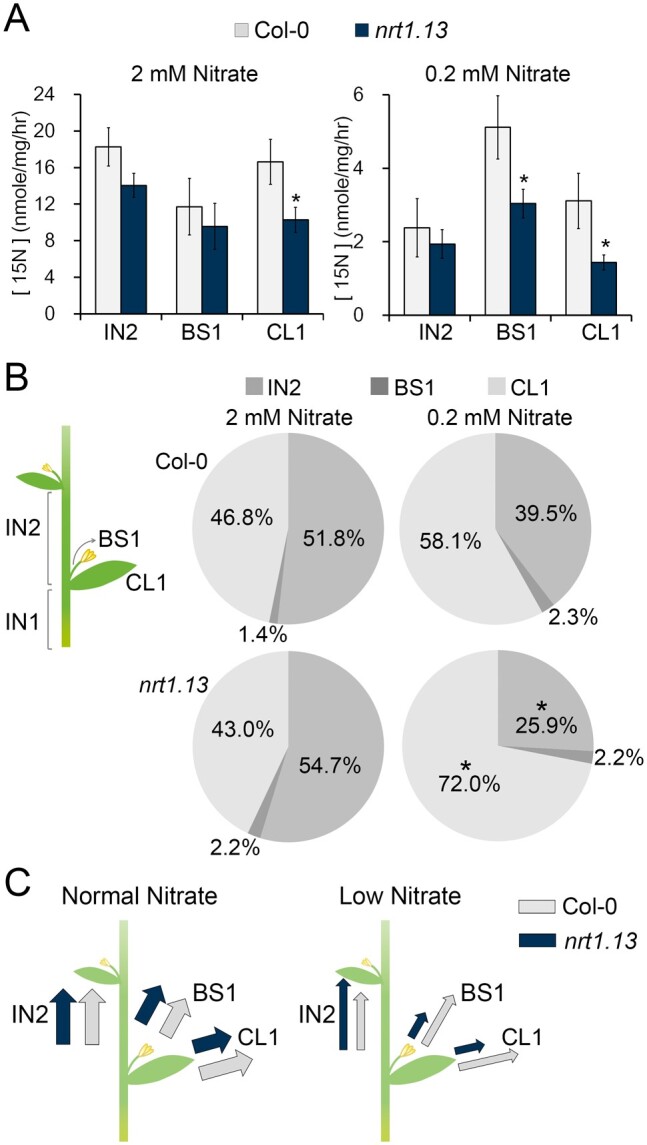
Nitrate allocation at nodes is modulated in *nrt1.13*. Nitrate allocation assay of plants supplied with 2 mM or 0.2 mM ^15^${\text{NO}}_{3}^{-}$. The ^15^N concentration of the basal cauline leaf (CL1), the stem of the basal cauline branch (BS1), and the internode between the basal node and second node (IN2) was determined as described in the Methods. Values are means of 21, 26, 17, and 28 independent plants of Col-0 (2 mM), *nrt1.13* (2 mM), Col-0 (0.2 mM), and *nrt1.13* (0.2 mM), respectively. (A) The ^15^N content in CL1, IN2, and BS1 of plants grown with 2 mM or 0.2 mM nitrate. Values are means ± SE (*, *P* < 0.05, Student’s *t*-test, compared with Col-0; Supplemental Data set 1). (B) The relative ratio of ^15^${\text{NO}}_{3}^{-}$ accumulation in CL1, BS1, and IN2. The relative ratio is calculated as the amount of ^15^N in each part divided by the total amount of ^15^N found in all three parts. (*, *P* < 0.05, Student’s *t*-test, compared with Col-0; Supplemental Data set 1). (C) Model of nitrate allocation in Col-0 (white arrow) and *nrt1.13* (grey arrow) under normal (2 mM) and low (0.2 mM) nitrate conditions.

*NRT1.13* is expressed at the nodes, so we were interested to determine whether *NRT1.13* can regulate nitrate distribution at nodes. To assess this, plants grown with normal (2 mM) or low (0.2 mM) nitrate were fed with ^15^${\text{NO}}_{3}^{–}$ at either 2 mM for one hour or 0.2 mM for two hours, and then, the ^15^N content of the stem segments above the nodes, as well as the branches and cauline leaves growing out of the nodes, was analyzed. We observed a major difference between Col-0 and *nrt1.13* in nitrate allocation under low nitrate. At high nitrate, cauline leaf ^15^N content was reduced to 62% that of the wild-type level (Figure 6A), but there was no difference for branches. At low nitrate, compared with Col-0, less ^15^N was allocated into both cauline leaves and branches; ^15^N content in cauline leaves and branches of *nrt1.13* were reduced to 46% and 59%, respectively, of the wild-type level (Figure 6A). When the relative distribution of ^15^N was compared in the three segments after the node—cauline leaf, branch, and internode between nodes 1 and 2 from the bottom—we observed that, under normal nitrate conditions, 53.2 ± 3.7% of ^15^N was allocated to the lateral parts including the cauline leaves and branches of Col-0 (Figure 6B), and a comparable ratio (57.0 ± 3.7%) was observed in *nrt1.13.* However, under low nitrate, the ratio of lateral ^15^N allocation was reduced to 39.5 ± 4.2% in Col-0 and further reduced to 28.0 ± 2.1% in *nrt1.13*. These data indicate that *NRT1.13* is important for lateral nitrate allocation at nodes and particularly under low-nitrate conditions.

### Node number and outgrowth are altered in *nrt1.13* in a nitrate concentration–dependent manner

**Figure 7:**
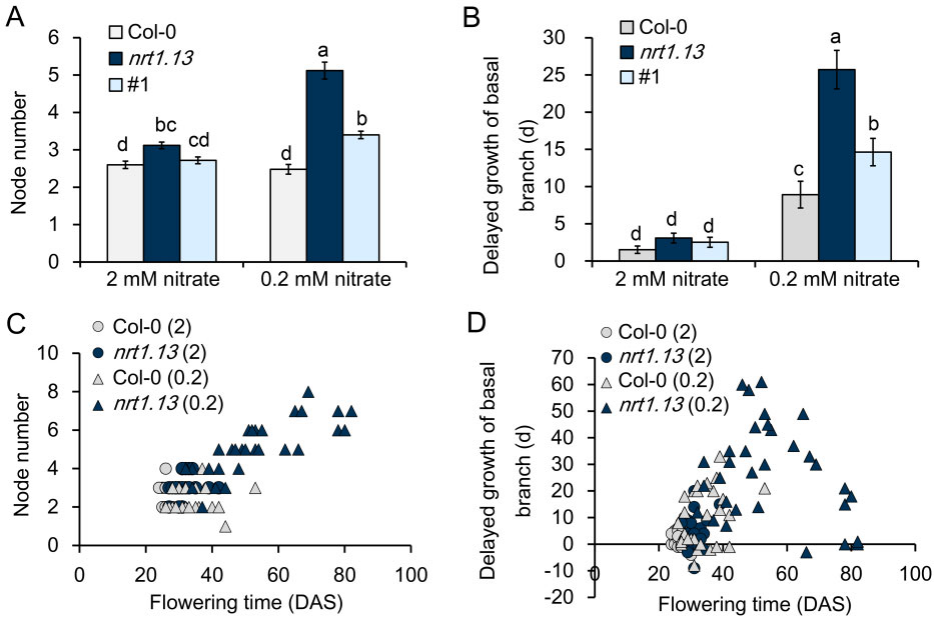
Branching pattern is altered in *nrt1.13*. (A) Node number of the primary inflorescence stem for plants grown under low (0.2 mM) and normal (2 mM) nitrate. Values are means ± SE of 25 independent plants. (B) Growth difference between basal and apical branches for plants grown at low and normal nitrate. The day when the apical and basal branch lengths reached 0.5 cm was recorded and compared. Data presented represent the delayed growth of basal branches compared to apical branches. Values are means ± SE of 25 independent plants. Statistical analysis in A and B comprised one-way ANOVA with a Tukey B *post hoc* test (*P*<0.05; Supplemental Data set 1). (C) and (D) Relationship between the phenotypes of flowering time and node number (C) or flowering time and delayed growth of basal branches (D) for plants grown under low or normal nitrate. Circles, normal nitrate (2 mM); triangles, low nitrate (0.2 mM); white, Col-0 plants; black, *nrt1.13* plants. Data from 28, 28, 30, and 32 plants of Col-0 (2 mM), Col-0 (0.2 mM), *nrt1.13* (2 mM), and *nrt1.13* (0.2 mM), respectively, are presented. The Pearson correlation coefficients (*r*) are 0.788 (*n*=124, *P*=0.000) between the flowering time and branch number and 0.467 (*n*=119, *P*=0.000) between the flowering time and outgrowth of basal branches (Supplemental Data set 1). Similar results were observed in another two experiments.

Since *NRT1.13* is expressed at the node (Figure 3B) and regulates lateral nitrate allocation (Figure 6), shoot architecture might be affected. The phenotypes of cauline branches in *nrt1.13* were examined under both normal- and low-nitrate conditions. Under normal concentrations of nitrate (2 mM), the number of nodes along the primary inflorescence stem was slightly increased in *nrt1.13* compared with Col-0 (Figure 7A). At low nitrate (0.2 mM), the difference between the wild type and mutant was more pronounced, as *nrt1.13* possessed twice as many nodes as Col-0 (Figure 7A). Thus, when the nitrate concentration was reduced to 0.2 mM, the node number of the primary inflorescence stem showed no change compared with that of normal nitrate in Col-0. By contrast, the node number was affected dramatically by nitrate supply in *nrt1.13*, increasing from an average of 3.1 under normal nitrate to 5.1 under low nitrate. In a complementation line, node number was reduced to a level comparable to that of Col-0 (Figure 7A). This result indicates that NRT1.13 can affect node formation, especially under low nitrate.

Another branch-related phenotype of *nrt1.13* is delayed outgrowth of basal branches. To quantify outgrowth of branches, we recorded the day when branch length reached 0.5 cm. Under normal nitrate conditions, outgrowth of basal branches compared with apical branches was delayed for an average of 2–3 days in both Col-0 and *nrt1.13* (Figure 7B, Supplemental Figure 6). In comparison, under low nitrate, outgrowth of basal branches was delayed for approximately nine days in Col-0 and a dramatic four weeks in *nrt1.13*. The branch growth phenotype of *nrt1.13* could be partially rescued in the complementation line (Figure 7B). This result shows that NRT1.13 can regulate branch outgrowth, and its influence is more significant on basal branches, particularly under low-nitrate conditions.

To determine the correlation between flowering and branch phenotypes, the flowering times of the wild-type and mutant plants were plotted against cauline branch number (Figure 7C) or outgrowth of basal branches (Figure 7D). A Pearson product-moment correlation coefficient was computed to assess the relationship between flowering time and branch phenotypes. There was a stronger positive correlation between flowering time and cauline branch number (*r* = 0.788, *P* = 0.000) compared with the correlation between flowering time and outgrowth of basal branches (*r* = 0.467, *P* = 0.000). As for the growth phenotypes, nitrate concentration exhibited little or no effect on node number of the primary inflorescence stem, flowering time, and basal branch outgrowth in Col-0. However, in *nrt1.13*, low nitrate led to late flowering, increased node number, and delayed basal branch outgrowth compared with normal-nitrate conditions. Thus, compared to wild type, *nrt1.13* is more sensitive to a reduction in nitrate supply, suggesting that *NRT1.13* plays an important role in acclimation to nitrate status, by which the reproductive transition and branch development are regulated.

## Discussion

### Role of NRT1.13 in nitrate regulation of plant development

CHL1 (NRT1.1/NPF6.3) of the NRT1 (NPF) family functions as a transceptor to monitor changes in nitrate concentration in soil and to regulate the primary nitrate response and lateral root development (Ho et al., 2009; Krouk et al., 2010). A proline residue in the cytosolic loop between the 10th and 11th transmembrane domains is well conserved in the family (Ho et al., 2009). Transport activity is abolished when this proline is mutated into leucine, but the sensing function is not affected. Interestingly, in *Arabidopsis*, only three members of the family (including NRT1.13) do not have the proline residue at the corresponding position (Supplemental Figure 1). As expected, NRT1.13 cannot transport nitrate when expressed in oocytes (Figure 1). However, when the serine in NRT1.13 is converted back to proline, nitrate transport ability can be detected, suggesting that NRT1.13 (which is located at the plasma membrane, Figure 2) can bind nitrate but cannot transport it across the membrane. *NRT1.13* is expressed in the parenchyma cells adjacent to xylem (Figure 3). When grown under normal nitrate concentrations, no dramatic visible growth phenotypes were observed in *nrt1.13*. By contrast, when grown under low nitrate, the mutant exhibited late flowering, increased cauline branch number, and arrested basal cauline branch outgrowth compared with Col-0 (Figures 4–7). Thus, these data suggest that NRT1.13 may function as a transceptor to monitor nitrate levels in the xylem and regulate the plasticity of shoot architecture. Nevertheless, we cannot completely exclude the alternative possibility that NRT1.13 without transport activity might interact with other nitrate transporters *in planta* to modulate nitrate distribution, leading to the observed developmental changes.

### The floral transition in *nrt1.13*

Mineral nutrients, particularly nitrogen and phosphate, are critical environmental cues regulating the floral transition (de Jong and Leyser, 2012; Vidal et al., 2014; Zhang et al., 2014). Compared with the vernalization, autonomous, photoperiod, gibberellin, and aging pathways, the mechanism of the nutrient-modulated floral transition pathway is less well characterized (de Jong and Leyser, 2012). Emerging evidence is revealing the role of nitrate in regulating the floral transition (Castro Marin et al., 2011; Kant et al., 2011; Liu et al., 2013; Yuan et al., 2016; Gras et al., 2018). These studies focused on how higher concentrations of nitrate/nitrogen delay flowering. When sufficiently broad ranges of nitrate concentrations have been examined simultaneously, it was observed that both extremely high and extremely low concentrations of nitrate delay flowering, with flowering time displaying a U-shaped response to nitrate concentrations (Lin and Tsay, 2017; Gras et al., 2018). In our study (Figure 4), the low-nitrate-induced flowering delay is more severe in the *nrt1.13* mutant and this *nrt1.13*-mediated flowering defect is nitrate concentration-dependent, indicating that *NRT1.13* participates in low-nitrate modulation of flowering time.

**Figure 8:**
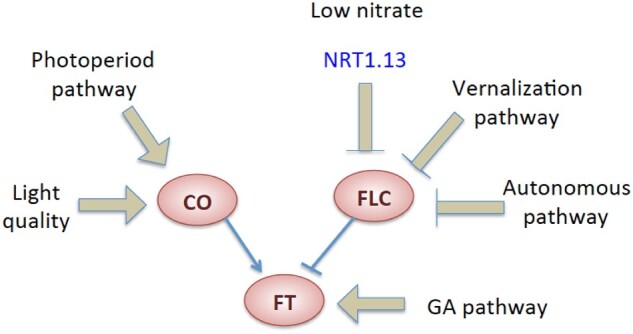
NRT1.13 is required to repress the expression of *FLC* in order to facilitate flowering under low-nitrate conditions. Apart from the vernalization and autonomous pathways, FLC is also a target of the nitrate-dependent regulation of flowering control.

Under the 0.2 mM nitrate condition, the delayed flowering mutant, *nrt1.13*, showed increased expression of the flowering repressor *FLC* and reduced expression of the positive regulator *LFY* (Figure 4). The role of FLC in NRT1.13-mediated low-nitrate flowering control is reinforced by the loss of the late-flowering phenotype in the *nrt1.13 flc* double mutant, suggesting that FLC could be an entry point for the low-nitrate response pathway (Figure 8). Kant et al. (2011) have shown that expression of *FLC* and *LFY*, but not *FT*, is changed upon altering nitrate concentrations, and Castro Marin et al. (2011) have shown that the flowering response to nitrate was abolished in *35S::FLC* mutants. Nevertheless, in the study by Castro Marin et al. (2011), *lfy* mutants still exhibited a nitrate-modulated change in flowering time, suggesting that *LFY* might not be the only downstream target of *FLC* in the nitrate regulatory pathway. Our study, together with these previous findings, suggests that the nitrate-modulated floral transition is mediated by regulating *FLC* and *LFY* expression and that *NRT1.13* is an additional player upstream of this regulatory pathway.

Castro Marin et al. (2011) showed for mutants defective in the autonomous pathway (*faw-1*, *fve-1*, *fy-1*), in the photoperiod pathway (*co2tt4*), in a target of the gibberellin pathway (*ft-*7), or for floral integrators (*fd-1*, *lfy*) that high nitrate still induces delayed flowering, suggesting that the nitrate regulation pathway is parallel to but independent of the autonomous, gibberellin, and photoperiod pathways. Consistent with that notion, we observed that expression of genes in these pathways showed no difference in the *nrt1.13* mutant. Nevertheless, a recent study reported that the floral repressors SCHLAFMUTZE (SMZ) and SCHNARCHZAPFEN (SNZ), which act downstream of gibberellin signaling, are required for the high-nitrate-elicited flowering delay (Gras et al., 2018). In addition, Yuan et al. (2016) identified a different nitrogen regulatory flowering pathway mediated by the blue-light receptor CRY1 (but not CRY2) and ferredoxin-NADP^+^-oxidoreductase (FNR1) in plants grown in the presence of both ammonium and nitrate (or ammonium alone) under long-daylight conditions. Our RNA-seq analysis shows that *SMZ*, *SNZ*, *CRY1*, and *FNR1* are not differentially expressed in the *nrt1.13* mutant. Since the studies by Yuan et al. (2016) and Gras et al. (2018) focus on high-N/nitrate-induced flowering delay, and our study investigates low-nitrate-induced flowering delay, the mechanisms responsible for different ranges of nitrate/nitrogen concentrations, that is for the left- and right-hand-sides of U-shaped flowering responses (Lin and Tsay, 2017), might be different.

In addition, it is known that nitrate has a strong influence on flowering time under short-day conditions, but has little effect under long days (Castro Marin et al., 2011). Under our experimental conditions of neutral daylight and with nitrate as the sole nitrogen source, the photoperiod pathway was not activated, so the FLC/NRT1.13 pathway might be more dominant. Likewise, trehalose-6-phostate (a proxy for carbohydrate status) regulates flowering via the induction of FT in the leaf under long-day conditions, but via the aging pathway at the shoot apical meristem independently of the photoperiod pathway (Wahl et al., 2013).

### Node number is regulated by nitrate availability in *nrt1.13*

When plants were grown under normal-nitrate concentrations (2 mM), the *nrt1.13* mutant exhibited an equivalent number of nodes along the primary inflorescent stem as wild type. However, under diminished nitrate (0.2 mM), the number of nodes in the mutant increased (Figure 7A). This nitrate-dependent pattern of altered node number is similar to the pattern of the flowering defect. Indeed, as shown in Figure 7, there was a strong positive correlation between flowering time and node number (*r* = 0.788). Consistent with this observation, several studies have already shown that both flowering time and shoot architecture are simultaneously altered in mutants of floral integrator genes or floral meristem identity genes, for example *lfy*, *tfl1*, *ft*, *tsf*, *ap1* (Shannon and Meeks-Wagner, 1991; Teo et al., 2014; Tsuji et al., 2015). Moreover, analysis of eight quantitative trait loci (QTLs) contributing to natural variation in reduced stem branching (RSB) has suggested that the flowering regulators are the candidate genes (Huang et al., 2012). Therefore, it is possible that the flowering and node number phenotypes of *nrt1.13* are regulated by the same pathway. In addition, the increased expression of *FLC* observed in *nrt1.13* (Figure 4F) might be simultaneously responsible for the late flowering and increased node number in the mutant (Figure 7A).

### Branch outgrowth and nitrate allocation in *nrt1.13*

Another defect we observed of *nrt1.13* is delayed outgrowth of the cauline branch, and particularly the basal branch, under low-nitrate conditions (Figure 7B, Supplemental Figure 6). In Col-0, low-nitrate delays branch outgrowth and this delay is more severe at the basal branch. This low-nitrate-induced delay of basal branch outgrowth was more pronounced in *nrt1.13*, suggesting that *NRT1.13* is involved in the nitrate-regulated basal branch outgrowth mechanism.

A similar effect of low nitrate on delayed basal bud activation (early termination of the basipetal activation sequence) has been reported in *Arabidopsis* and *Rosa hybrida* (de Jong et al., 2014; Furet et al., 2014). In addition to nitrogen deficiency, phosphate deficiency can also affect outgrowth of shoot branches (Umehara et al., 2010; Kohlen et al., 2011; Drummond et al., 2015). Analyses of branching responses to nutrients in several hormone mutants have suggested that cytokinin, strigolactone, and auxin might participate in or interact with nutrient signaling to regulate branch outgrowth (Umehara et al., 2010; Kohlen et al., 2011; de Jong et al., 2014; Drummond et al., 2015; Muller et al., 2015). Interestingly, in decapitated pea plants, bud release occurs before changes in auxin content and it is correlated better with sugar accumulation, suggesting that sugar demand rather than auxin is the primary regulator of bud activation (Mason et al., 2014). Thus, more and more evidence reveals the important role of nutrients in regulating shoot architecture.

Expression levels of *NRT1.13* are higher in the basal node than in the apical node (Figure 3C). Consistent with this expression pattern, branch outgrowth inhibition by low nitrate was more pronounced in the basal branches of *nrt1.13* (Figure 6B, Supplemental Figure 6). We examined expression levels of several hormone marker genes in the basal node, but no significant change was detected in *nrt1.13.* Therefore, further evidence is required to determine whether hormones are involved in the shoot architecture defect of *nrt1.13*. Our ^15^N allocation analysis showed that at the basal node, less ^15^N was allocated laterally to the cauline leaf and branch in *nrt1.13*, particularly under low-nitrate conditions (Figure 6). Like the branch outgrowth defect, the lateral ^15^N allocation defect was more evident at low nitrate, so the outgrowth defect may be due to reduced nitrate allocation to the branch. Since our functional study showed that NRT1.13 cannot transport nitrate directly (Figure 1), the allocation defect may be due to an indirect effect of NRT1.13 on either expression or activation of some unknown transporters through post-transcriptional regulation or protein–protein interactions.

### The function of NRT1.13 without the conserved proline residue

All described functional transporters in the NRT1 (NPF) family contain the conserved proline residue between transmembrane domains 10 and 11 (Supplemental Figure 1). Like NRT1.13, SP1 (OsNPF4.1) does not have the conserved proline residue and shows no transport activity for tested substrates, including nitrate, dipeptides, histidine, glutamate, proline, citrulline, ammonium, dicarboxylates, or hexoses (Li et al., 2009). In rice, *sp1* shows a defect in basal panicle elongation and exhibits the short-panicle phenotype. Shoot development is known to be regulated by plant hormones and, recently, several studies have shown that substrates of NRT1 (PTR) members can be extended from nitrate and peptide to auxin, abscisic acid, glucosinolate, gibberellin, and jasmonoyl-isoleucine (JA-Ile) (Sugiura et al., 2007; Krouk et al., 2010; Kanno et al., 2012; Nour-Eldin et al., 2012; Leran et al., 2014; Chiba et al., 2015). Although NRT1.13 has shown no ABA, GA, or JA-Ile transport ability (Kanno et al., 2012; Chiba et al., 2015) and the proline residue in CHL1 is important for auxin transport (Krouk et al., 2010), the possibility for NRT1.13 without the corresponding proline residue to transport hormones might be low but cannot be completely ruled out.

Our functional analysis in Xenopus oocyte shows that wild-type NRT1.13 cannot transport nitrate, but the S487P mutation recovered nitrate transport activity (Figure 1). Single amino acid substitutions in a protein may alter protein stability or targeting. Nevertheless, when we injected the same amount of cRNA into Xenopus oocytes, the protein accumulation levels of wild-type NRT1.13 and S487P were similar (Supplemental Figure 2), indicating that it is more likely that the substitution of S487 with proline does not affect the protein stability. In addition, both wild-type NRT1.13 and S487P-GPF localize in the plasma membrane upon transient expression in mesophyll protoplasts (Figure 2). Therefore, the lack of nitrate transport activity of wild-type NRT1.13 is not due to changes in protein stability or targeting and, instead, it is more likely that wild-type NRT1.13 is a defective nitrate transporter. Thus, unless an as-yet unknown partner protein, present only *in planta* but not in Xenopus oocytes, is required to restore the conformation and transport activity of NRT1.13, the available data suggest that the transport activity might not be required for the function of NRT1.13 to regulate flowering and the plasticity of the shoot development.

The crystal structure of CHL1 suggests that the highly conserved proline reside at 492 is important for structural coordination of the two helices constituting transmembrane domains 10 and 11 (Parker and Newstead, 2014; Sun et al., 2014). It has been suggested that E476 on transmembrane domain 10, which forms an intracellular gate with K164 in the outward open conformation and supports substrate binding of the histidine residue (H356) in the inward open conformation (Parker and Newstead, 2014; Sun et al., 2014), may be important for the transition between different conformations. Substituting the proline with other residues in *chl1-9*, SP1, and NRT1.13 may hamper the conformational change required for substrate transport.

NRT1.13 cannot transport nitrate but it can bind nitrate, and the phenotypes of *nrt1.13* are nitrate concentration-dependent, suggesting that NRT1.13 may monitor changing nitrate concentrations and regulate shoot development. Nitrate acquired from soil is transported from roots to various tissues via the xylem. NRT1.13 expressed in xylem parenchyma cells could thereby monitor the nitrate supply in the xylem and regulate flowering, branch initiation, and basal branch outgrowth. The defective phenotypes of *nrt1.13* are more severe under low-nitrate conditions, indicating that NRT1.13 is required for low-nitrate acclimation. At low nitrate, NRT1.13 may activate some salvage processes, for example by enhancing lateral allocation at the nodes to facilitate branch outgrowth and by attenuating FLC expression to control flowering, and thereby overcome the nutrient shortage. Study of CHL1 has indicated that external nitrate can be monitored at root surfaces to regulate gene expression and root development (Ho et al., 2009; Krouk et al., 2010). Our study of NRT1.13 suggests that internal nitrate can be monitored near the xylem to regulate shoot architecture.

## Materials and methods

### Plant material, growth conditions, and phenotype analysis

*Arabidopsis thaliana* Columbia-0 ecotype (Col-0) was used as the wild-type control. T-DNA mutants *nrt1.13* (SAIL_258_H05) and *nrt1.13-2* (WiscDsLoxHs064_12G) were obtained from the Arabidopsis Biological Resource Center (http://abrc.osu.edu/) (Sessions et al., 2002). The T-DNA insertion was confirmed by Genomic PCR using F, R, and LB primers listed in Supplemental Table 2. For complementation lines *P_NRT1.13_:NRT1.13-GFP/nrt1.13 #1* and *#2*, the genomic fragment including a 2-kb upstream promoter and *NRT1.13* coding region was amplified by PCR using the 2kb-F primer and *NRT1.13*-R primer (sequences listed in Supplemental Table 2), cloned in-frame with GFP in the binary vector pMDC107 (Curtis and Grossniklaus, 2003), and then introduced into *nrt1.13.* For *P_NRT1.13_:GUS*, the genomic fragment from the 2-kb promoter to the middle of the second exon was amplified by PCR using the 2kb-F primer and exon2-R primer, cloned into vector pMDC163 (Curtis and Grossniklaus, 2003), and then introduced into Col-0.

Most plants were grown on the hydroponic system from Araponics (http://www.araponics.com/) in a growth chamber (12-h light/12-h dark, light source: Philips Lifemax Cool White, 70–80 µmol m^−2^ s^−1^, 23°C). After cold treatment, seeds were germinated on rock wool in water and hydroponic buffer was applied at day 3. The hydroponic buffer, including basal nutrients (1 mM KH_2_PO_4_/K_2_HPO_4_, 2 mM MgSO_4_, 1 mM CaCl_2_, 0.1 mM FeSO_4_-EDTA, 50 µM H_3_BO_3_, 12 µM MnSO_4_·2H_2_O, 1 µM ZnCl_2_, 1 µM CuSO_4_·5H_2_O, 0.2 µM NaMoO_4_·2H_2_O, 0.05% (w/v) MES, adjusted to pH 5.7 with KOH) and 2 mM KNO_3_ or 0.2mM KNO_3_ plus 1.8 mM KCl, was renewed three times per week. For tissue expression studies, plants were grown with 0.2 mM KNO_3_. For floral relative gene expression analysis, plants were grown with 0.2 mM KNO_3_ and then harvested at 25 days after germination. For the nitrate allocation study, plants were grown with 2 or 0.2 mM KNO_3_ until the basal branch reached 5 mm in length. Delayed growth of basal branches was calculated as the day after sowing required for the basal branch to reach 5 mm in length, minus the day required for the apical branch to reach 5 mm in length.

### Nitrate uptake assay in *Xenopus* oocytes and nitrate allocation assay in plants

*NRT1.13* wild-type cDNA was amplified by PCR using the primer pairs F (SmaI) and R (BamHI), and cloned into the oocyte expression vector pGEMHE (Liman et al., 1992). The mutated *NRT1.13* (*S487P*) cDNA was generated by rolling-cycle PCR using primer pairs F (S487P) and R (487P). The pGEMHE *NRT1.13* and pGEMHE *NRT1.13* (*S487P*) plasmids were linearized with NheI. 50-ng capped mRNAs, *in vitro* transcribed using mMESSAGE mMACHINE kits (Ambion), and 50-nl water were injected into *Xenopus* oocytes based on modified processes described previously (Tsay et al., 1993; Wang and Tsay, 2011). After a 2-day incubation in ND96 solution (96 mM NaCl, 2 mM KCl, 1 mM MgCl_2_, 1.8 mM CaCl_2_, and 5 mM HEPES, pH 7.4) containing 0.005% (w/v) gentamycin, the oocytes were then incubated for 2.5 h in nitrate uptake buffer containing 5 mM or 150 µM K^15^NO_3_ and 220 or 230 mM mannitol, respectively, as well as 0.3 mM CaCl_2_, 10 mM MES-Tris pH 5.5, before being rinsed with ND96 solution and dried at 80°C for 24 h. The retained ^15^N was measured using a continuous-flow isotope ratio mass spectrometer coupled to a carbon nitrogen elemental analyzer (ANCA-GSL MS; PDZ Europa) as previously described (Hsu and Tsay, 2013).

For the nitrate allocation assay, plants were incubated in hydroponic buffer containing 2 mM K^15^NO_3_ or 0.2mM K^15^NO_3_ plus 1.8 mM KCl for 1 or 2 h, respectively, and washed with 0.1 mM CaSO_4_ three times. The different tissues collected were dried at 80°C. After the dried weight was measured, the amount of ^15^${\text{NO}}_{3}^{–}$ in different tissues was analyzed using ANCA-GSL MS. The relative ^15^N content in different tissues was calculated and defined as ^15^N_tissue_/(^15^N_CL1_+^15^N_IN2_+^15^N_BS1_) X 100%.

### Subcellular localization in protoplasts

*NRT1.13* cDNA was amplified by PCR using the primer pair CDS-F and CDS-R and cloned in-frame with GFP in the modified 326-GFP vector (Lee et al., 2001) with an added Gateway cassette. This fusion construct or the control vector was isolated by a Qiagen plasmid kit and transiently expressed in *Arabidopsis* protoplasts following the protocol described by Sheen (2001). Protoplasts were isolated from rosette leaves of 3- to 4-week-old plants grown on soil. After incubation in W5 solution under light for 17 h, fluorescent cells were imaged as described by Wang and Tsay (2011).

### GUS staining and GFP localization

GUS staining was performed as previously described (Wang and Tsay, 2011; Hsu and Tsay, 2013) with slight modification. *P_NRT1.13_:GUS* plants were grown in the soil under continuous light as described in Almagro et al. (2008) and harvested on the indicated day, and then incubated in X-Gluc staining solution (50 mM sodium phosphate pH 7.0, 0.05% Triton X-100, 1 mM potassium ferrocyanide, 1 mM potassium ferricyanide, and 1 mM 5-bromo-4-chloro-3-indoyl-b-D-glucuronide) for 10 h at 37°C. After three washes, tissues were cleared in a graded series of ethanol. GUS staining was then visualized by AxioImager-Z1 (Zeiss). For sections, tissues were embedded in LR White medium-grade resin (London Resin Company). Then, 3 µm semi-fine sections were cut, mounted on glass slides, and counterstained with periodic acid-Schiff reagent (Sigma-Aldrich).

For the NRT1.13-GFP protein localization study, *P_NRT1.13_:NRT1.13-GFP/nrt1.13 #1* and *nrt1.13* were grown hydroponically with 0.2 or 2 mM KNO_3_ and then harvested on the indicated day. The nodes were embedded in 5% (w/v) agarose dissolved in water and cut into 120-µm sections with a Vibratome Series1000 (Technical Products International). The slices of agarose were mounted on slides and then observed using a confocal Zeiss LSM780 microscope as previously described (Hsu and Tsay, 2013).

### RNA extraction, library construction, and sequencing

Total RNA was extracted from the shoot using TRIzol reagent (Gibco BRL) and subjected to quality control with an Agilent 2100 Bioanalyzer. Libraries were prepared using a TruSeq Stranded mRNA LT set A/B Sample Preparation kit (Illumina, USA) with three biological replicates, each from a single plant, for each treatment and 2 µg of total RNA as input. Polyadenylated RNA was isolated using poly-T oligo-attached magnetic beads and fragmented using divalent cations under elevated temperature (94°C) for 8 min. The size-enriched (250-300 bp) RNA fragments were subjected to first-strand cDNA synthesis using random primers and SuperScript 2 (Invitrogen), and then second-strand cDNA synthesis using DNA polymerase I and RNase H. After end-repair and A-tailing, indexing adaptors were ligated and the DNA fragments with adaptors on both ends were purified and amplified by 12 cycles of PCR. After validation and quantification using a KAPA library quantification kit (Peqlab), we pooled 24 libraries and sequenced them on an Illumina NextSeq500 platform using a NextSeq 500 High output v2 (150 cycles) sequencing kit to generate high-quality paired-end reads of 75 bp in length.

### RNA-seq data analysis

All reads were trimmed and quality-filtered using CLC Genomics Workbench 10 (Qiagen), with settings of removal of low-quality sequence (limit = 0.01), no ambiguous nucleotides allowed, and removal of reads smaller than 10 nucleotides. The trimmed reads were mapped to the TAIR10 genome using the RNA-seq mapping algorithm implemented in CLC Genomics Workbench 10 and allowing only unique mapping with a maximum of two mismatches. We obtained more than 23 million reads mapped in pairs per library. The raw data with GEO accession number GSE162242 have been deposited in the NCBI Gene Expression Omnibus. The expression level of each gene was calculated as Count Per Millilon (CPM). Using CLC Genomics Workbench 10 (Qiagen) for analysis, genes with CPM ≥ 2 in all 24 samples, fold-change ≥ 1.8, and *P* value ≤ 0.05 were considered differentially expressed.

### RT-quantitative pCR

Total RNA was extracted from the indicated tissues using TRIzol reagent (Gibco BRL). The first-strand cDNAs were synthesized using oligo(dT) primers and ImProm-II reverse transcriptase (Promega). Quantitative PCR was performed using a LightCycler^®^480 System (Roche) programed for 10 min at 95°C as pre-incubation, and then 40–65 cycles of 10 sec at 95°C, 5 sec at 59°C, and 11 sec at 72°C. The primer sets used for RT-qPCR are listed in Supplemental Table 2.

### Accession numbers

Sequence data from this article can be found in the Arabidopsis Genome Initiative databases under the following accession numbers: At1g33440 (*NRT1.13*), At5g10140 (*FLC*), At5g15840 (*CO*), At1g65480 (*FT*), At2g45660 (*SOC1*), At5g61850 (*LFY*), At1g13440 (GAPDH), At4g05320 (UBQ10), At4g40040 (*HIS3*), At1g08090 (*NRT2.1*), and At1g37130 (*NIA2*). The RNAseq raw data are deposited in the NCBI Gene Expression Omnibus under the GEO accession number GSE162242.

## Supplemental data

**Supplemental Figure 1**. Amino acid sequence alignment of *SP1* in rice and *NRT1/PTR* genes in *Arabidopsis*.

**Supplemental Figure 2**. Protein expression levels in *Xenopus* oocytes.

**Supplemental Figure 3.** Binding isotherms for nitrate to NRT1.13 reveal that purified NRT1.13 protein can bind nitrate.

**Supplemental Figure 4.** The *nrt1.13-2* mutant also exhibits a late-flowering phenotype under low-nitrate conditions.

**Supplemental Figure 5.** High-affinity and low-affinity nitrate responses are not altered in the *nrt1.13* mutant.

**Supplemental Figure 6.** Apical and basal branch growth of plants grown under normal (2 mM) and low (0.2 mM) nitrate indicated as the days after bolting when branch length is over 0.5 cm.

**Supplemental Table 1.** Expression of flowering genes in *nrt1.13*.

**Supplemental Table 2.** The primer sets used for the constructs, Genomic DNA PCR, RT-PCR, and RT-qPCR used in this paper.

**Supplemental Methods.** Protein expression levels in Xenopus oocytes; Microscale thermophoresis binding assay; Primary nitrate responses.

**Supplemental Data set 1.** Results of statistical analyses.

## Supplementary Material

koab051_Supplementary_Data

## Notes

H.-Y.C., S.-H.L., L.-H.C., J.-J. W., Y.-C.L., and Y.-F.T. conceived and designed the experiments. H.-Y. C. performed the experiments of Figure 1, 3, 4C∼F, 6, and 7, supplemental Figure 2 and 6, S.-H.L. performed the experiments of Figure 2, supplemental Figure 4 and 5, and RNAseq analysis. L.-H.C. performed the initial study of characterizing *nrt1.13* mutant and generating the *P_NRT1.13_:GUS* and *P_NRT1.13_:NRT1.13-GFP* lines, and the experiments of Figure 2, 4A, 4B, and Supplemental Figure 1. J.-J.W. performed the experiments of Figure 5. Y.-C.L. performed the experiments of Supplemental Figure 3. H.-Y.C., S.-H.L., and Y.-F.T. wrote the manuscript.

The author responsible for distribution of materials integral to the findings presented in this article in accordance with the policy described in the Instructions for Authors (https://academic.oup.com/plcell) is: Yi-Fang Tsay (yftsay@gate.sinica.edu.tw)
