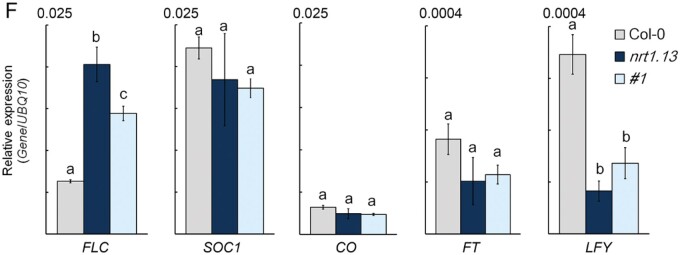# Corrigendum to: Potential transceptor AtNRT1.13 modulates shoot architecture and flowering time in a nitrate-dependent manner

**DOI:** 10.1093/plcell/koab172

**Published:** 2021-07-23

**Authors:** Hui-Yu Chen, Shan-Hua Lin, Ling-Hsin Cheng, Jeng-Jong Wu, Yi-Chen Lin, Yi-Fang Tsay

*The Plant Cell*, koab051, https://doi.org/10.1093/plcell/koab051

During the conversion of black-and-white figures into color figures, the authors misplaced a sub-figure of LFY expression in Figure 4, panel F. A corrected version of the figure panel appears below and the original article has been updated. The authors apologize for the error.

**Figure:**