# Supplementary material for: Potential transceptor AtNRT1.13 modulates shoot architecture and flowering time in a nitrate-dependent manner
Source: Plant Cell. 2021 Feb 12;33(5):1492–505. doi: 10.1093/plcell/koab051 (PMC8254489; doi:10.1093/plcell/koab051)
Supplement: koab051_Supplementary_Data [file koab051_supplementary_data.zip › tpc.00771.2020-s01.pdf]

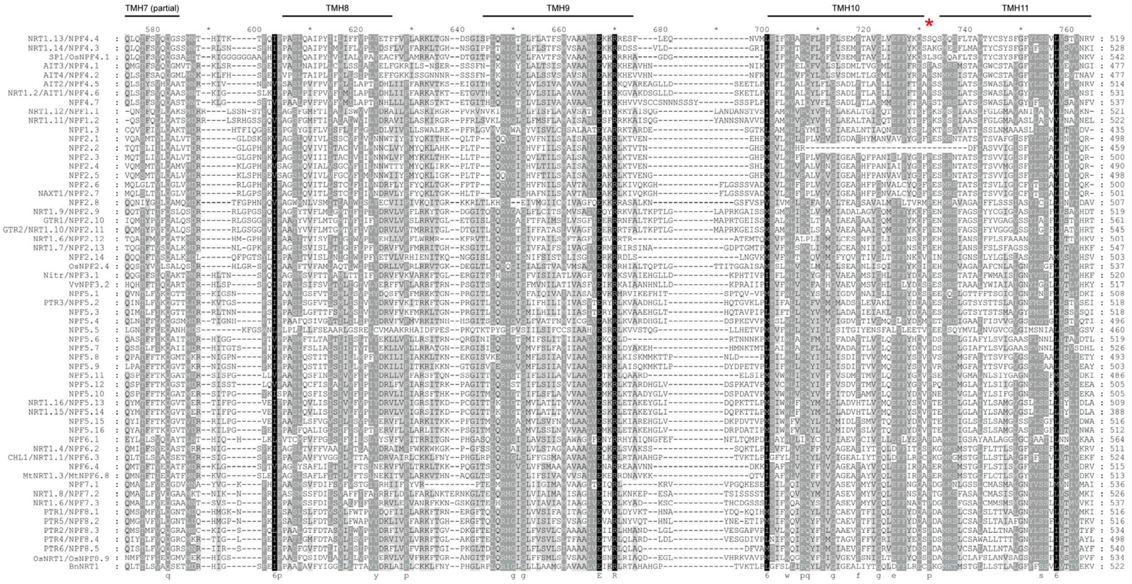

Supplemental Figure 1. Amino acid sequence alignment of *SP1* in rice and *NRT1/PTR* genes in *Arabidopsis*. (Supports Figure 1.)

The amino acid alignment was performed using the ClustalX 2.0 program. The red star indicates Pro492 in CHL1/NPF6.3. The transmembrane helix regions were assigned according to the CHL1 crystal structure (Parker and Newstead, 2014; Sun et al., 2014).

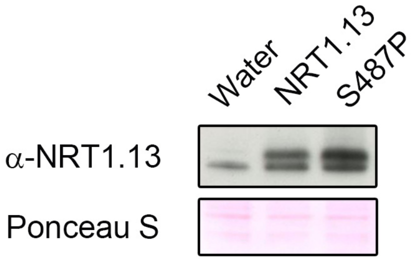

Supplemental Figure 2. Protein expression levels in Xenopus oocytes. (Supports Figure 1.)

Total protein of four injected oocytes were loaded in each line. Equal protein loading was confirmed by Ponceau S staining, and protein levels of NRT1.13 were analyzed by hybridizing to NRT1.13 antibodies.

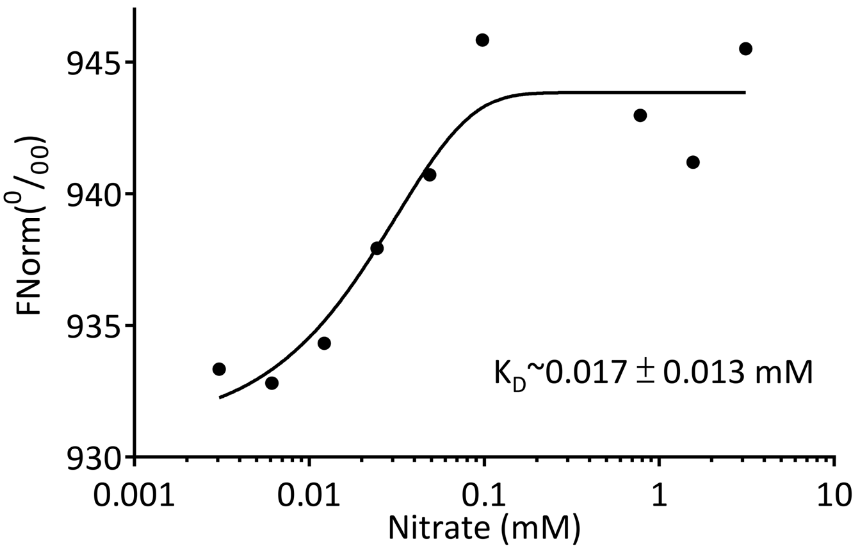

Supplemental Figure 3. Binding isotherms for nitrate to NRT1.13 reveal that purified NRT1.13 protein can bind nitrate. (Supports Figure 1.)

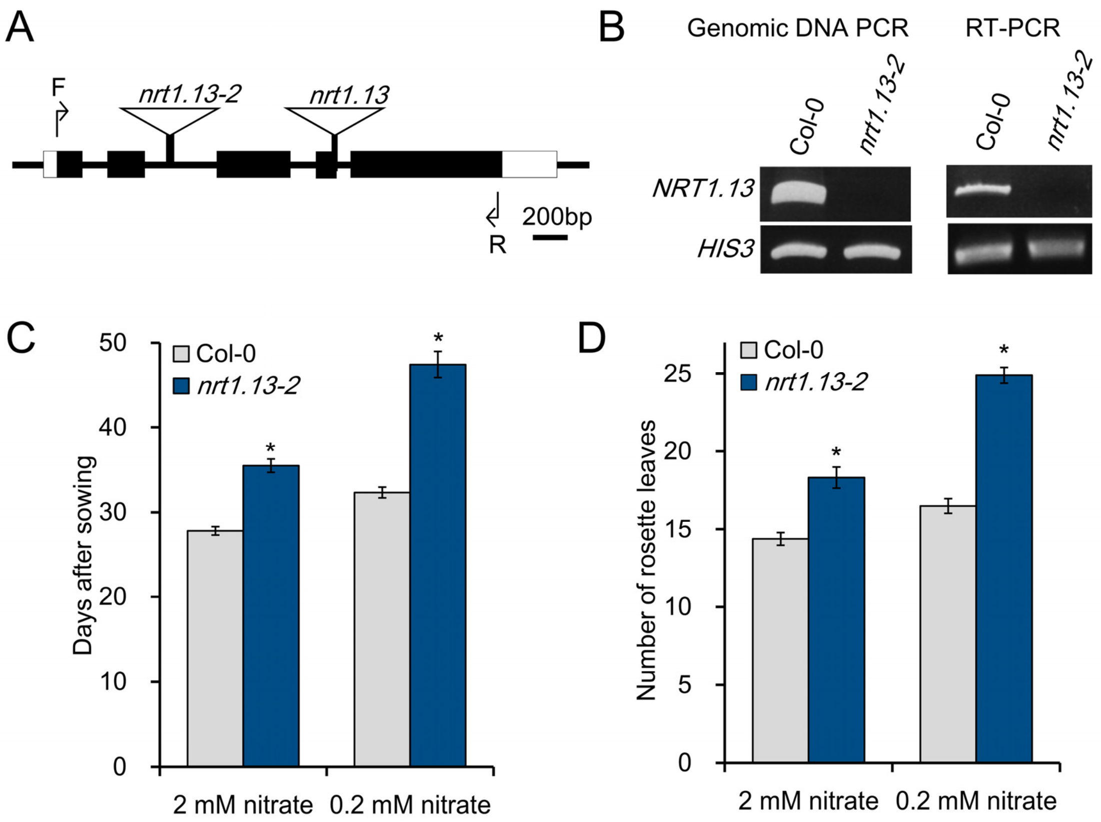

Supplemental Figure 4. The *nrt1.13-2* mutant also exhibits a late-flowering phenotype under low-nitrate conditions. (Supports Figure 4.)

(A) Schematic of the T-DNA insertion sites of the *nrt1.13-2* mutant. In *nrt1.13-2*, the T-DNA was inserted into the second intron. Black boxes, coding region; white boxes, untranslated region; F and R, the forward and reverse primers, respectively, used for genomic DNA PCR and RT-PCR.
(B) Genomic DNA PCR and RT-PCR analyses of *nrt1.13-2*. *HIS3* was used as an internal control for genomic and RT-PCR.
(C) and **(D)** The flowering time of plants grown under normal (2 mM) or low (0.2 mM) nitrate are indicated as days after sowing (C) or the number of rosette leaves at bolting (D). Values are means ± SE of 19~28 independent plants. (*, P < 0.05, Student's *t*-test, compared with Col-0; Supplemental Data set 1).

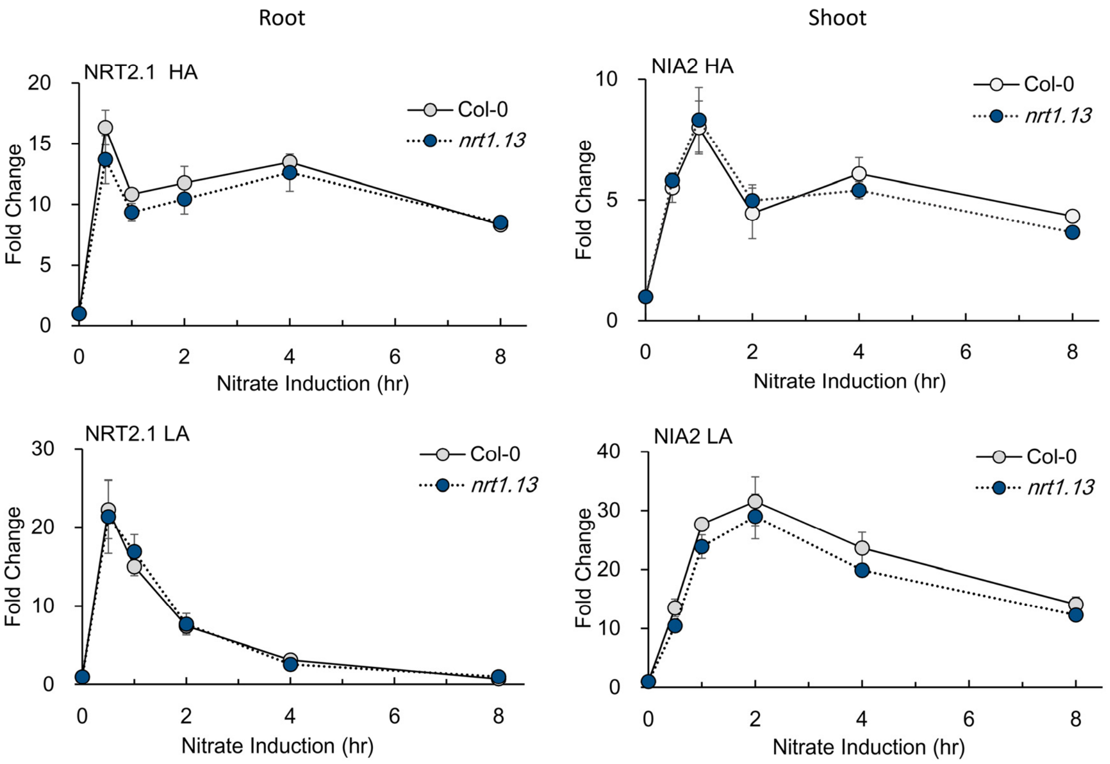

Supplemental Figure 5. High-affinity and low-affinity nitrate responses are not altered in the *nrt1.13* mutant. (Supports Figure 4F.)

Plants were grown in a magenta box supported with 12.5 mM ammonia succinate medium (pH6.5) for 8 days, then pretreated with 12.5 mM ammonia succinate medium (pH5.5) for 16 hours, and finally refreshed for another 3 h. For the high affinity response, 0.25 mM KNO_3 medium (pH5.5) was used (HA, upper panels), whereas for the low affinity response (LA, bottom panels), 10 mM KNO_3 medium (pH5.5) was used. The root and shoot of seedlings were separated and collected for further analysis. Data was normalized to the zero point and are expressed as fold-change, with each point representing the mean from four repeats.

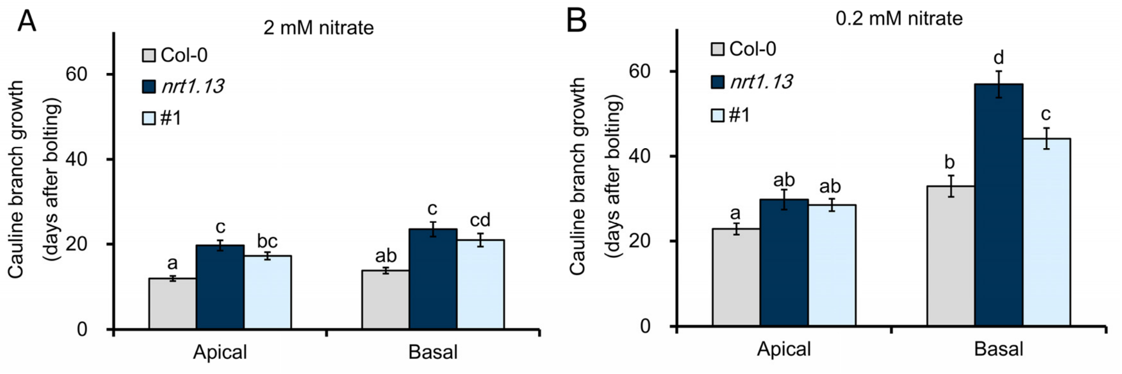

Supplemental Figure 6. Apical and basal branch growth of plants grown under normal (2 mM) **(A)** and low (0.2 mM) **(B)** nitrate indicated as the days after bolting when branch length is over 0.5 cm. (Supports Figure 7.)

Values are means ± SE of 25 independent plants. Statistical analysis comprised one-way ANOVA with a Tukey B post-hoc test ($p<0.05$; Supplemental Data set 1). Similar results were obtained in three independent experiments.

Supplemental Table 1. Expression of flowering genes in *nrt1.13*

		16 DAG				25 DAG			
		0.2		2		0.2		2	
Locus	Gene	FC	P	FC	P	FC	P	FC	P
**Integrator**									
At5g10140	FLC	1.97	0.00	1.40	0.00	2.75	0.00	1.78	0.00
At2g45660	SOC1	-1.37	0.00	-1.13	0.11	-1.24	0.03	-1.06	0.46
At4g24540	AGL24	1.16	0.05	1.13	0.09	1.06	0.42	1.04	0.42
**Vernaliztion**									
At5g17690	LHP1	-1.02	0.75	-1.02	0.78	1.03	0.77	-1.04	0.49
At3g18990	VRN1	1.08	0.30	1.07	0.17	1.06	0.44	1.03	0.71
At4g16845	VRN2	-1.02	0.77	-1.04	0.45	-1.00	0.97	-1.00	0.97
At4g00650	FRI	-1.04	0.72	1.04	0.67	-1.04	0.69	1.04	0.68
At5g16320	FRL1	-1.01	0.97	1.00	0.99	-1.09	0.51	1.03	0.83
At4g29830	VIP3	1.08	0.30	-1.15	0.01	1.01	0.86	1.01	0.93
At5g61150	VIP4	1.04	0.59	1.01	0.90	-1.03	0.68	1.04	0.45
At3g12810	PIE1	-1.04	0.62	1.01	0.91	-1.02	0.74	-1.11	0.04
At4g15880	ESD4	-1.05	0.54	-1.03	0.68	1.00	0.97	1.07	0.36
**Autonomous**									
At4g02560	LD	1.03	0.69	1.01	0.87	-1.00	0.98	-1.02	0.75
At4g16280	FCA	1.11	0.16	1.00	0.95	1.03	0.66	-1.00	1.00
At3g10390	FLD	-1.02	0.83	-1.05	0.36	1.05	0.50	-1.09	0.27
At3g04610	FLK	1.14	0.07	1.04	0.48	-1.03	0.68	-1.06	0.21
At2g43410	FPA	1.05	0.46	-1.00	0.99	-1.03	0.68	1.10	0.07
At2g19520	FVE	1.05	0.49	-1.03	0.49	-1.09	0.23	-1.01	0.91
At5g13480	FY	-1.03	0.72	-1.08	0.16	-1.08	0.29	-1.10	0.21
**Photoperiod pathway**									
At1g68050	FKF1	-1.03	0.68	-1.12	0.02	-1.05	0.51	-1.02	0.67
At5g62430	CDF1	1.02	0.88	1.11	0.30	1.02	0.83	-1.05	0.53
At5g15840	CO	-1.15	0.33	-1.13	0.34	-1.18	0.19	-1.02	0.86
At2g32950	COP1	-1.01	0.88	1.03	0.58	-1.02	0.79	1.01	0.86
At1g04400	CRY2	-1.03	0.67	1.03	0.56	1.01	0.91	-1.00	0.97
At4g08920	CRY1	1.06	0.37	1.00	0.92	1.01	0.88	1.07	0.14
At1g22770	GI	1.03	0.66	-1.12	0.02	-1.02	0.73	-1.11	0.03
At1g09570	PHYA	1.02	0.79	-1.00	0.98	-1.04	0.58	1.01	0.89
At2g18790	PHYB	-1.02	0.83	1.04	0.46	1.01	0.84	-1.04	0.39
At2g39250	SNZ	1.12	0.13	1.14	0.01	1.18	0.02	1.04	0.59
At2g46340	SPA1	-1.12	0.13	1.02	0.75	-1.03	0.70	1.04	0.42
At3g15354	SPA3	1.10	0.18	1.12	0.03	1.06	0.39	1.09	0.07
At1g53090	SPA4	-1.08	0.30	1.15	0.08	1.08	0.35	1.03	0.72
At3g07650	COL9	-1.03	0.64	1.01	0.91	1.00	0.98	1.02	0.75
**Ambient temperature**									
At3g33520	ARP6	-1.05	0.49	1.08	0.31	1.04	0.58	-1.04	0.58
At1g77080	MAF1	1.21	0.01	1.05	0.50	1.01	0.92	1.04	0.45
At5g65050	MAF2	1.27	0.00	1.11	0.04	1.01	0.91	-1.03	0.57
At2g43010	PIF4	-1.04	0.60	-1.04	0.48	-1.05	0.48	1.08	0.10
At2g22540	SVP	1.09	0.21	-1.03	0.59	1.03	0.66	1.04	0.40
**GA**									
At1g14920	GAI	1.06	0.39	-1.00	0.95	1.02	0.73	1.05	0.36
At3g05120	GID1A	-1.12	0.14	-1.03	0.67	-1.03	0.74	-1.02	0.70
At5g27320	GID1C	1.31	0.01	1.03	0.69	1.05	0.68	1.27	0.01
At5g56860	GNC	-1.10	0.19	1.04	0.61	-1.08	0.27	-1.06	0.43
At4g26150	GNL	-1.12	0.29	1.06	0.47	-1.37	0.00	-1.06	0.52
At2g01570	RGA	1.01	0.84	-1.04	0.48	-1.03	0.73	1.05	0.36
At3g11540	SPY	1.04	0.61	1.03	0.60	1.04	0.62	-1.06	0.26
**Aging**									
At2g33810	SPL3	-1.21	0.10	-1.03	0.74	-1.19	0.13	-1.08	0.34
At1g53160	SPL4	-1.19	0.26	-1.26	0.11	-1.51	0.00	-1.48	0.00
At2g42200	SPL9	1.01	0.91	-1.01	0.87	-1.16	0.17	-1.10	0.22
At3g57920	SPL15	-1.19	0.25	-1.14	0.35	-1.36	0.09	-1.35	0.02

FC: fold-change of gene differential expression comparing *nrt1.13* with wild type, positive value means up-regulation and negative value means down-regulation in the *nrt1.13*; P: P-value.

Supplemental Table 2. The primer sets used for the constructs, Genomic DNA PCR, RT-PCR, and qRT-qPCR used in this paper.

Construct for $P_{NRT1.13}$:*NRT1.13-GFP/nrt1.13*	
2kb-F	5’-CACCGATTATGCGGTCGGTAGAGCATCAC-3’
NRT1.13-R	5’-AATTCTAGGCTTCTCCGTCGTGG-3’
Construct for $P_{NRT1.13}$:*GUS*	
exon2-R	5’-GATCGCCATCATCTCGAAAGCTTGG-3’
pGEMHE-NRT1.13 and NRT1.13(S487P)	
F (SmaI)	5’- CCCGGGTCACCATGGATGTTCATG-3’
R (BamHI)	5’-CCCGGATCCTCACCCCCCAATTCTAGGCTTCTCCGTCGTGGAATCC-3’
F (S487P)	5’- GTGGAGTTTTTTACAAACAGTCTCCACAGAGTATGCAATCTTTTCTC-3’
R (S487P)	5’- GAGAAAAGATTGCATACTCTGTGGAGACTGTTTGTAAAAAAACTCCAC-3’
NRT1.13-326GFP	
CDS-F	5’-CACCATGGATGTTCATGATCTATCTG-3’
CDS-R	5’-AATTCTAGGCTTCTCCGTCGTGG-3’
Genomic DNA PCR and RT-PCR	
LB	5’-TAGCATCTGAATTTCATAACCAATCTCGATACAC-3’
F (*NRT1.13*)	5’-ATGGATGTTCATGATCTATCTG-3’
R (*NRT1.13*)	5’-TCAATGAAGCTAAAAGCCAG-3’
F (*HIS3*)	5’-AACCACTGGAGGAGTCAAGA-3’
R (*HIS3*)	5’-CAATTAAGCACGTTCTCCTCTG-3’
RT-qPCR	
F (*GAPDH*)	5’-TTGGTGACAACAGGTCAAGCA-3’
R (*GAPDH*)	5’-AAACTTGTCGCTCAATGCAATC-3’
F (*NRT1.13*)	5’-AGAAGGTTGGTTAGGCGATAATGA-3’
R (*NRT1.13*)	5’-GGACGGATCACACGAATACCAT-3’
F (*UBQ10*)	5’-GGCCGTACGTTGGCTGATT-3’
R (*UBQ10*)	5’-CCCAGTCAACGTCTTAACGAAAA-3’

F (*FLC*)	5’-ATGGTTCACACTATGAGCTAC-3’
R (*FLC*)	5’-CAACATGAGTTCGGTCTTC-3’
F (*CO*)	5’-GGACTCACTACAACGACAAT-3’
R (*CO*)	5’-CTGAGTTGTGTTACTGTTATCATCT-3’
F (*FT*)	5’-TCCAAGTCCTAGCAACCC-3’
R (*FT*)	5’-AAACAATATAAACACGACACGATG-3’
F (*SOC1*)	5’-AAATATGAAGCAGCAAACATGAT-3’
R (*SOC1*)	5’-TGCTCGAATACATTTGACACT-3’
F (*LFY*)	5’-GTCCAGACAATTGCTAAAGACC-3’
R (*LFY*)	5-‘GCTTCTTCGTCTAGGCAG-3’
F (*NRT2.1*)	GGCCGTACGTTGGCTGATT
R (*NRT2.1*)	CCCAGTCAACGTCTTAACGAAAA
F (*NIA2*)	GGCTCATGTCTCAGTACC
R (*NIA2*)	GGTATTGCTCTGCCCA

Supplemental Methods

Protein expression levels in *Xenopus* oocytes.

Protein from oocytes was analyzed by immunoblotting using anti-NRT1.13 antibody (1:2000). The anti-NRT1.13 rabbit polyclonal antibodies were generated and purified using synthetic peptide corresponding to the a.a. 6~23 of NRT1.13. by LTK BioLaboratories (http://www.ltk.com.tw).

Microscale thermophoresis binding assay

The gene encoding NRT1.13 was cloned into a GFP^{His} fusion yeast expression vector pDDGFP_LEU2D. NRT1.13 was expressed and purified according to the method described previously (Parker and Newstead, 2014). Fluorescence labeling of NRT1.13 was performed following the protocol for NHS-coupling of the dye NT647 (NanoTemper Technologies, Munich, Germany) to lysine residues. Binding was calculated for NRT1.13 using microscale thermophoresis. Nitrate at concentrations ranging from 3 µM to 3.13 mM was incubated with 5 nM of purified GFP-tagged protein for 5 min in assay buffer (20 mM Bis-Tris pH 5.5, 0.03% DDM). The sample was loaded into NanoTemper glass capillaries and microthermophoresis was carried out using 5% LED power and 40% MST in a NanoTemper Monolith $NT.115^{pico}$ system. K_D values were calculated using the mass action equation in the NanoTemper software.

Primary nitrate responses

Plants were grown in hydroponic culture for 8 days in NO_3^--free medium using NH_4^+ as the sole N source, then treated with fresh medium overnight and again for an additional 3 h, before being exposed for the indicated time to high affinity (HA) 0.25mM KNO_3 or low affinity (LA) 10 mM KNO_3 (Ho et al, 2009).
