# Supplementary material for: Potential transceptor AtNRT1.13 modulates shoot architecture and flowering time in a nitrate-dependent manner
Source: Plant Cell. 2021 Feb 12;33(5):1492–505. doi: 10.1093/plcell/koab051 (PMC8254489; doi:10.1093/plcell/koab051)
Supplement: koab051_Supplementary_Data [file koab051_supplementary_data.zip › tpc.00771.2020-s05.pdf]

Potential Transceptor AtNRT1.13 Modulates Shoot Architecture and Flowering Time in a Nitrate-Dependent Manner

Hui-Yu Chen, Shan-Hua Lin, Ling-Hsin Cheng, Jeng-Jong Wu, Yi-Chen Lin and Yi-Fang Tsay

Corresponding author: Yi-Fang Tsay yftsay@gate.sinica.edu.tw

Review timeline:

TPC2018-RA-00884	Submission received:	Nov. 20, 2018
	1st Decision:	Jan. 3, 2019 *manuscript declined*
**TPC2020-RA-00771D**	Submission received:	Sept. 17, 2020
	1st Decision:	Oct. 26, 2020 *Accept with minor revisions*
**TPC2020-RA-00771DR1**	1st Revision received:	Nov. 30, 2020
	2nd Decision:	Dec. 7, 2020 *revision requested*
**TPC2020-RA-00771DR2**	2nd Revision received:	Jan. 1, 2021
	3rd Decision:	Jan. 9, 2021 *acceptance pending, sent to science editor*
	Final acceptance:	Feb. 3, 2021

REPORT: (The report shows the major requests for revision and author responses. Minor comments for revision and miscellaneous correspondence are not included. The original format may not be reflected in this compilation, but the reviewer comments and author responses are not edited, except to correct minor typographical or spelling errors that could be a source of ambiguity.)

TPC2018-RA-00884 1st Editorial decision – *declined* Jan. 3, 2019

Your submission has been evaluated by members of the editorial board as well as expert reviewers in your field, and we regret to inform you that we are not able to recommend publication of this manuscript. We have not made this decision lightly. We have had input from multiple scientists, and have solicited post-review comments as well. Our present policy is to offer streamlined decisions and to not advise on the direction of the work by requesting extensive modifications or substantial additional experiments.

All reviewers found your manuscript interesting and the topic appropriate for the journal, but all requested substantial new data to improve the manuscript prior to acceptance. All argued that the molecular mechanism by which NRT1.13 acts is not clear, as emphasised specifically by reviewer 2. Major issues include whether NRT1.13 can transport nitrate or not, for example can it transport nitrate when oocytes are preloaded with nitrate? Can it bind nitrate? Also, could its substrate be altered from NO3- to Cl-, as suggested by reviewer 3? There were also issues about the complementation and genetics, emphasised by reviewer 1. For example, analysing one mutant allele and showing incomplete complementation is not fully convincing and could the S487P fully complement the mutant phenotype including the flowering time? Based on these comments and others, we decided in the post-review consultation to reject this manuscript. However, we would be interested in a new version that includes substantial new data to answer the reviewers' concerns. Otherwise, we suggest that you send this version to another journal. The reviewers point out a number of areas in which the work could be strengthened, which may be helpful to you as you continue your project or revise your manuscript for submission elsewhere. If you decide to complete the story and resubmit to The Plant Cell, it will be evaluated as a new submission subject to full assessment by the editorial board and if sent for external review the same set of reviewers will be chosen.

-- Reviewer comments:

Reviewer #1 (Comments for the Author):

Chen et al report that a protein belonging to NPF family, AtNRT1.13, functions potentially as a nitrate transceptor in modulating flowering time and shoot architecture. This is indeed an attractive important finding for understandings

how nitrate regulating growth period and architecture and final yield. I have the following major concerning before the manuscript could be accepted for publication:

1） The authors showed that partial complementation of nrt1.13 with wild type NRT1.13 (Fig.4D, Fig.S3). They concluded that NRT1.13 affects floral transition in a nitrate-dependent manner. It is very interesting to know the phenotype of complementation of nrt1.13 by expressing mutated NRT1.13 with S487P which might have dual functions in nitrate transport and sensing. Will it show complete recovery of the mutant and loss of the nitrate-independent manner?

2） For concluding that FLC could be the critical player in the NRT1.13-controlled pathway, it is also important to know if NRT1.13 regulates FLC expression directly or indirectly. I would like to suggest the enhanced expression (over-expression) of FLC in nrt1.13 to further confirm that NRT1.13 negatively regulates FLC.

3） In the manuscript, the author mentioned that transceptor NRT1.13 is able to bind nitrate, is this conclusion made based on experiment results or just a speculation? The authors clarified NRT1.13 as a transceptor for nitrate, but did not provide evidence for its function on sensing nitrate. Is the nrt1.13 mutant still able to sense nitrate? The defect phenotype might also due to the loss of its function on sensing nitrate. Since mutated NRT1.13 (S487P) could restore nitrate uptake, would it also rescue the flowering defect? How does mutated NRT1.13 respond to altered nitrate conditions?

4） One important issue to be addressed is the function of NRT1.3 at which level of nitrate. The change from Ser487 to proline increased nitrate uptake activity just in high N (5mM) not in low N (Fig.1). It is clear that WT NRT1.13 is unable to uptake nitrate, but nrt1.13 mutant exhibited defect on nitrate allocation in cauline leaf and cauline branch at low nitrate level, which is very interesting. The authors assumed that it might be attributed to the indirect effect of NRT1.13 on the expression or activation of nitrate transporters. However, no nitrate transporter exhibited altered expression level in the RNAseq dataset. Did authors check any well-known nitrate transporters, e.g. CHL1?

5） The author has previously reported that flowering time of Arabidopsis is delayed under either low or high condition (Lin and Tsay, 2017). In this manuscript, the authors only compared the flowering time of WT and nrt1.13 mutant under normal (2 mM) and low nitrate (0.2 mM) conditions. What would be the flowering-related phenotype when the mutant is treated with high nitrate? To decipher whether flowering phenotype of NRT1.13 is nitrate-dependent, the authors should also check the flowering time of the mutant under high nitrate concentration (~10 mM).

6） Only when a near fully complementation was observed can the readers be convinced that the phenotype of one T-DNA insertion line represents the gene function. In the study, the authors just showed one mutant line (nrt1.13), while the complementary lines only partially (less than 50% based on Fig.4CD; Fig.7; Fig.S3; S4) rescue the nrt1.13 phenotype, and this should be discussed by the authors.

Minor comments:

7） In Figure 2, the authors showed the PM localization of NRT1.13, but in Figure 3K, the translation fusion of the NRT1.13 seems not localize at PM? In addition, how about the localization of mutated NRT1.13 (S487P)? It would be interesting to check it.

8） For the expression pattern of NRT1.13 promoter, the GUS signal in Figure 3, it is necessary to indicate the sampling time (growth stage) and the N regime (nitrate concentration, duration).

9） The author mentioned in Abstract and result that nrt1.13 showed no severe phenotype grown at normal nitrate condition, however, in Fig.4C, only the phenotype at low nitrate supply was shown. I would like to see their difference of the mutant and WT at low and high nitrate conditions.

10） Page 6, line 112, "supplementary Figure 1" changed to "supplementary Figure 2";

11） Page 8, line 157, "Figure 4C" changed to "Figure 4C, D";

12） Line 195: "expression of LFY was significantly increased in the nrt1.13 mutant (Figure 4E).". Actually, LFY expression was decreased in the mutant.

13） Replace the Supplemental figure 3 with Figure 4D or just add Fig.S3 into Fig.4 since flowering time is the critical parameter determined in this manuscript.

14） Supplementary Figure 4: Suggest to separate Apical and basal branch growth (BA, BB) to two sub-figures.

Reviewer #2 (Comments for the Author):

In this work Chen et al reports the role of NRT1.13/NPF4.4. The interesting feature of this protein is that it displays a "natural mutation" on the residue equivalent to P492 in CHL1. The wild type version of NRT1.13 does not transport nitrate in oocytes, when its S487P version does. Its mutation in plants triggers nitrate related phenotypes reminiscent to a transceptor activity. According to its localization and transcriptomic studies of the mutant, authors propose that NRT1.13 is able to monitor in planta NO3- concentrations and control nitrate allocation and flowering via a FLC-dependent pathway.

This work is well performed and observations are of great interest for the Nitrate/Nitrogen community.

The only remark I have is that it is difficult to draw a molecular mechanism by which NRT1.13 is involved in the sensing of NO3- and how NRT1.13 influences Nitrate allocation/flowering.

I would suggest answering this questions by the following experiments. These are suggestions. Performing all the experiments is not mandatory for me to support the publication of this work. But having some hints concerning the molecular mechanism is needed, to my opinion.

-1) It might be interesting to see if the S487P version if able to complement the flowering phenotypes. This will give us an idea of Nitrate transport contribution to the phenotype.

-2) Is CHL1 WT and P492L versions under NRT1.13 promoter able to complement the phenotype? Same idea as above.

-3) Is Primary Nitrate Response (PNR) impacted in nrt1.13 mutant? It would be nice to perform QPCR on PNR reporters in the conditions of Figure 5. This will give insights into the role of nrt1.13 in controlling a well-defined NO3-related signaling pathway.

-4) Is pCHL1:NRT1.13 (WT and S487P) is able to complement chl1 mutants for PNR?

-5) Is NRT1.13 is really not a Nitrate transporter? Please provide a range of concentrations for Nitrate uptake in oocytes, since the partial complementation of the phenotype in planta could be explained by a partial nitrate transport activity not seen in oocytes.

-6) NRT1.13/NPF4.4 has already been shown as not being able to transport NO3- (Leran et al Scientific Report), but belongs to a sub-family with GA and ABA transporters. GAs are known flowering controllers. Thus, as discussed by the authors, it would be nice to test GA transport by NRT1.13...

-7) Is NRT1.13 a sort of "natural dominant negative" that will impact the activity of another NRT1/NPF? Can you co-inject CHL1 and NRT1.13 for instance and test nitrate transport? Or look for a NPF being co-expressed in the parenchyma cells and test interaction for a) nitrate transport [should explain the nitrate allocation phenotype] or b) hormone transport [could explain the flowering phenotype]...

-Minor: p6,lines 115-117. Authors state that NRT1.13 is able to bind NO3-. To my understanding of the work nothing support this conclusion. This should be rephrase or data supporting this conclusion should be provided.

-Finally and most importantly, to my opinion NRT1.13 could probably be an active NO3- transporter in plants, in peculiar conditions not reflected in oocyte experiments. Indeed, recent report has shown that P492L version of CHL1 is able to transport NO3- when oocytes are pre-loaded with NO3- (Noguero et al, https://www.biorxiv.org/content/early/2018/02/26/244467). The underlying hypothesis is that CHL1-P492L perceives

internal-NO3- at higher concentrations (with lower affinity) to switch the transporter activity on. If NRT1.13 is a "natural version" of that kind, NO3- concentration in plant cells should be enough to activate NRT1.13 NO3- transport activity and this would explain the nrt1.13 mutant phenotype... I think authors need to test this hypothesis that could clearly explain why nrt1.13 have a NO3- related phenotypes in planta. This can be made by preforming the same experiments as Noguero et al or following point 1,2,4 above.

Again this work is of very good quality but we are just missing information/hypothesis about the actual mechanism that will explain the link between oocytes and in planta observations. Any substantial explanation(s) following my suggestions above (or any other insight) should be provided for publication in high impact journal.

Reviewer #3 (Comments for the Author):

In this manuscript Chen et al show that AtNRT1.13 does not exhibit nitrate transport activity but can act as a transceptor. This is likely due to the exchange of proline 487 with a serine. Deletion mutants for AtNRT1.13 exhibit a pronounced phenotype under nitrogen starvation: delayed flowering, an increased node number as well as retarded branch outgrowth and nitrate allocation to nodes. This is a careful study describing the phenotype, cellular and tissue localization as well as effects on flowering time genes under nitrogen starvation of AtNRT1.13.

Nevertheless, there are a few points that should be clarified:

- The authors claim that AtNRT1.13 bind nitrate. Although this seems likely this is just a hypothesis. The best would be if they could proof such a binding, but I am aware that this is not easy. However at least a much more careful formulation should be used. Could it be that AtNRT1.13 interacts was a nitrate transporting NRT and by doing so modulates its transport activity. If this cannot be excluded the authors should discuss also this possibility.

- The difference in nitrate content in the mutant implies that the transport activity for nitrate is reduced. The authors mention this fact in one sentence in the discussion, but I would like to get a deeper discussion. Is no other NRT deregulated? Or could this altered transport activity be due to an interaction of AtNRT1.13 with other NRTs as mentioned above?

- For CLCs it has been shown that a similar P to Ser mutations shifts the substrate specificity from NO3- to Cl-. I strongly suggest to investigate whether AtNRT1.13 does not act (also) as a chloride transporter/channel. Loss of such an activity may also have an impact on nitrate partitioning. In case AtNRT1.13 can transport chloride, chloride contents should be measured.

TPC2020-RA-00771D	Submission received	Sept. 17, 2020

Reviewer comments on previously declined manuscript and **author responses:**

We thank you for your time and effort in handling our manuscript. Your insightful comments and those of the reviewers have helped us to improve the manuscript. According to your combined suggestions, we have modified this manuscript by:

1) Adding the flowering time of a second mutant, *nrt1.13-2*, in Supplementary Figure 4, which confirms that NRT1.13 is involved in flowering control;

2) Adding the microscale thermophoresis nitrate binding assay of NRT1.13 protein in Supplementary Figure 3 to confirm that NRT1.13 can bind nitrate;

3) Adding the result of the primary nitrate response of the *nrt1.13* mutant in Supplementary Figure 5, which indicates that NRT1.13 is not involved in the primary nitrate response and, therefore, NRT1.13 is functionally different from NRT1.1 (CHL1);

4) Adding a photo of the *nrt1.13* mutant grown under normal N conditions in Figure 4C to confirm that the flowering defect is more severe under low N conditions.

In addition, in our "Response to reviewers", we present:

5) A nitrate uptake analysis of oocytes preloaded with nitrate (Figure L1), showing that internal nitrate does not

affect the nitrate uptake abilities of NRT1.13 and NRT1.13 S487P;

6) Flowering time of plants grown over a wide range of nitrate concentrations (Figure L2), showing that NRT1.13 has a more profound function under low nitrate in terms of flowering control;

7) A subcellular localization study of NRT1.13 S487P (Figure L3), showing that both NRT1.13 and NRT1.13 S487P localize to the plasma membrane.

All reviewers found your manuscript interesting and the topic appropriate for the journal, but all requested substantial new data to improve the manuscript prior to acceptance. All argued that the molecular mechanism by which NRT1.13 acts is not clear, as emphasised specifically by reviewer 2.

Major issues include whether NRT1.13 can transport nitrate or not, for example can it transport nitrate when oocytes are preloaded with nitrate?

Response: To address this concern, we injected nitrate into oocytes to attain a final concentration of ~10 mM KNO_3. Under this condition, NRT1.13 still shows no nitrate uptake activity compared with water-injected oocytes, indicating that internal nitrate does not impact the properties of NRT1.13 or the S487P mutation.

Can it bind nitrate?

Response: We now present two lines of evidence to demonstrate that NRT1.13 can bind nitrate. The first is to show the nitrate transport activity of NRT1.13-S487P. Since residue S487 does not lie in the substrate binding pocket, the fact that NRT1.13-S487P exhibits nitrate transport activity indicates that the substrate binding pocket of wild type NRT1.13 can recognize nitrate. A respective description about the position of S487P has been added in line 125 of the revised manuscript. Secondly, to further confirm that wild type NRT1.13 can bind nitrate, we performed a microscale thermophoresis binding assay using purified NRT1.13 protein. As shown in new Supplementary Figure 3, the resulting binding isotherms reveal that NRT1.13 can bind nitrate.

Also, could its substrate be altered from NO3- to Cl-, as suggested by reviewer 3?

Response: According to the structure of CLCec1, AtCLCa residue P160 that is responsible for substrate selectivity lies in the transmembrane region and, more specifically, within the transport channel. Based on the CHL1 crystal structure, NRT1.13 residue S487, which corresponds to P492 in the CHL1 structure, is located in the cytosolic loop and, consequently, not in the substrate binding pocket. Therefore, it is unlikely that substrate selectivity will be changed by Pro-to-Ser mutation in NRT1.13. A respective description about the position of residue P492 has now been added to the revised manuscript in line 112.

There were also issues about the complementation and genetics, emphasised by reviewer 1. For example, analysing one mutant allele and showing incomplete complementation is not fully convincing and could the S487P fully complement the mutant phenotype including the flowering time?

Response: To confirm the role of NRT1.13 in flowering regulation, we have isolated another NRT1.13 mutant, *nrt1.13-2*, and observed that it also displays a similar flowering phenotype (new Supplementary Figure 4). The partial complementation might be due to lower expression of the transgene, and introducing the S487P mutation might induce a similar problem.

Based on these comments and others, we decided in the post-review consultation to reject this manuscript.

However, we would be interested in a new version that includes substantial new data to answer the reviewers' concerns. Otherwise, we suggest that you send this version to another journal. The reviewers point out a number of areas in which the work could be strengthened, which may be helpful to you as you continue your project or revise your manuscript for submission elsewhere. If you decide to complete the story and resubmit to The Plant Cell, it will be evaluated as a new submission subject to full assessment by the editorial board and if sent for external review the same set of reviewers will be chosen.

Reviewer #1

The authors showed that partial complementation of *nrt1.13* with wild type NRT1.13 (Fig.4D, Fig.S3). They concluded that NRT1.13 affects floral transition in a nitrate-dependent manner. It is very interesting to know

the phenotype of complementation of *nrt1.13* by expressing mutated NRT1.13 with S487P which might have dual functions in nitrate transport and sensing. Will it show complete recovery of the mutant and loss of the nitrate-independent manner?

Response: To confirm the role of NRT1.13 in flowering regulation, we have isolated another NRT1.13 mutant, nrt1.13-2, and observed that it also displays a similar flowering phenotype (new Supplementary Figure 4). The partial complementation might be due to lower expression of the transgene, and introducing the S487P mutation might induce a similar problem.

For concluding that FLC could be the critical player in the NRT1.13-controlled pathway, it is also important to know if NRT1.13 regulates *FLC* expression directly or indirectly. I would like to suggest the enhanced expression (over-expression) of *FLC* in *nrt1.13* to further confirm that NRT1.13 negatively regulates *FLC*.

Response: *NRT1.13* is expressed in xylem parenchyma cells, and FLC for flowering control seems to be critical in companion cells. Therefore, cell-to-cell communication might be involved. Establishing the molecular link between NRT1.13 and FLC is one of our future research goals. Rather than overexpressing *FLC* in all cells driven by the 35S promoter, we have used a *flc* mutant to determine if FLC is involved in the NRT1.13 pathway. The phenotype of the *flc nrt1.13* double mutant indicates that functional FLC is required for the late flowering phenotype of *nrt1.13*.

In the manuscript, the author mentioned that transceptor NRT1.13 is able tobind nitrate, is this conclusion made based on experiment results or just a speculation? The authors clarified NRT1.13 as a transceptor for nitrate, but did not provide evidence for its function on sensing nitrate. Is the *nrt1.13* mutant still able to sense nitrate? The defect phenotype might also due to the loss of its function on sensing nitrate. Since mutated NRT1.13 (S487P) could restore nitrate uptake, would it also rescue the flowering defect? How does mutated NRT1.13 respond to altered nitrate conditions?

Response: Our previous studies have shown that the P492 residue in CHL1, which is located in the cytosolic loop between transmembrane domains 10 and 11, is required for nitrate transport but not nitrate sensing. Substrate binding sites lie in the transmembrane region. When residue S487 of NRT1.13 (corresponding to P492 in CHL1) is converted to Proline, the conformational change required for transport is rescued so it can transport nitrate. This scenario indicates that the nitrate binding pocket in the transmembrane domain of NRT1.13 is functional. To further confirm that NRT1.13 can bind nitrate, we performed a microscale thermophoresis binding assay using purified NRT1.13 protein. As shown in new Supplementary Figure 3, the resulting binding isotherms reveal that NRT1.13 can bind nitrate.

The function of NRT1.13 in nitrate sensing was inferred from altered nitrate- dependent behavior in the *nrt1.13* mutant. There are multiple nitrate-sensing mechanisms in the shoot, with NRT1.13 being one of them. The severe late flowering phenotype of the *nrt1.13* mutant under low nitrate conditions indicates that NRT1.13 can sense nitrate and play a significant role in alleviating the impact of low nitrate by, for example, repressing the expression of *FLC* and facilitating lateral nitrate transport.

One important issue to be addressed is the function of NRT1.3 at which level of nitrate. The change from Ser487 to proline increased nitrate uptake activity just in high N (5mM) not in low N (Fig.1). It is clear that WT NRT1.13 is unable to uptake nitrate, but *nrt1.13* mutant exhibited defect on nitrate allocation in cauline leaf and cauline branch at low nitrate level, which is very interesting. The authors assumed that it might be attributed to the indirect effect of NRT1.13 on the expression or activation of nitrate transporters. However, no nitrate transporter exhibited altered expression level in the RNAseq dataset. Did authors check any well-known nitrate transporters, e.g. CHL1?

Response: We have two transcriptome datasets, one for the leaf and one for the node. In both datasets, there is either no or very slight (non-significant) change for all NRT1 and NRT2 genes. For example, *CHL1* expression is 1.34-fold that of wild type level in the node of the *nrt1.13* mutant, but exhibits no difference in the leaf. In addition, expression of *NPF8.4*, which does not transport nitrate (our preliminary result), is 50% that of wild type level. We acknowledge that changes in posttranscriptional activation of transport and expression in a few critical cells cannot be revealed by transcriptomic analyses. A respective discussion of the potential mechanisms has now been added in line 453 of the revised Discussion.

The author has previously reported that flowering time of Arabidopsis is delayed under either low or high condition (Lin and Tsay, 2017). In this manuscript, the authors only compared the flowering time of WT and *nrt1.13* mutant under normal (2 mM) and low nitrate (0.2 mM) conditions. What would be the flowering-related phenotype when the mutant is treated with high nitrate? To decipher whether flowering phenotype of NRT1.13 is nitrate-dependent, the authors should also check the flowering time of the mutant under high nitrate concentration (~10 mM).

Response: When we tested a wide range of nitrate concentrations, flowering time displays a U-shaped curve for both wild type and *nrt1.13* mutant plants, with 2 or 5 mM forming the base of the curve. At higher nitrate including the base of the curve, flowering of *nrt1.13* was delayed by only 2~3 days compared to wild type, whereas it was delayed by ~7 days at low nitrate. Thus, flowering of *nrt1.13* is delayed more dramatically at low nitrate, indicating that *nrt1.13* has a more profound function at low nitrate in controlling flowering.

Only when a near fully complementation was observed can the readers be convinced that the phenotype of one T-DNA insertion line represents the gene function. In the study, the authors just showed one mutant line (*nrt1.13*), while the complementary lines only partially (less than 50% based on Fig.4CD; Fig.7; Fig.S3; S4) rescue the nrt1.13 phenotype, and this should be discussed by the authors.

Response: To confirm the role of NRT1.13 in flowering regulation, we have isolated another NRT1.13 mutant, *nrt1.13-2*, and it also displayed a similar flowering phenotype (new Supplementary Figure 4).

Minor comments:

In Figure 2, the authors showed the PM localization of NRT1.13, but in Figure 3K, the translation fusion of the NRT1.13 seems not localize at PM? In addition, how about the localization of mutated NRT1.13 (S487P)? It would be interesting to check it.

Response: The resolution of Figure 3K is insufficient to determine the subcellular localization of NRT1.13-GFP. Like wild type NRT1.13, mutated NRT1.13-S487P-GFP also presents a plasma membrane localization when transiently expressed in protoplasts.

For the expression pattern of NRT1.13 promoter, the GUS signal in Figure 3, it is necessary to indicate the sampling time (growth stage) and the N regime (nitrate concentration, duration).

Response: Agreed. We have now added this information to the figure legend and Material and Methods.

The author mentioned in Abstract and result that *nrt1.13* showed no severe phenotype grown at normal nitrate condition, however, in Fig.4C, only the phenotype at low nitrate supply was shown. I would like to see their difference of the mutant and WT at low and high nitrate conditions.

Response: As requested, we have now added an image of plants grown under normal nitrate conditions to Figure 4C.

Page 6, line 112, "supplementary Figure 1" changed to "supplementary Figure 2";

Response: Apologies, now corrected.

Page 8, line 157, "Figure 4C" changed to "Figure 4C, D";

Response: Apologies, now corrected.

Line 195: "expression of LFY was significantly increased in the nrt1.13 mutant (Figure 4E).". Actually, LFY expression was decreased in the mutant.

Response: Apologies, now corrected.

Replace the Supplemental figure 3 with Figure 4D or just add Fig.S3 into Fig.4 since flowering time is the critical parameter determined in this manuscript.

Response: As requested, Supplemental Figure 3 has now been moved into Figure 4.

Supplementary Figure 4: Suggest to separate Apical and basal branch growth (BA, BB) to two sub-figures.

Response: As suggested, apical and basal branch growth are now separated into two sub-figures in Supplementary Figure 6.

Reviewer #2

In this work Chen et al reports the role of NRT1.13/NPF4.4. The interesting feature of this protein is that it displays a "natural mutation" on the residue equivalent to P492 in CHL1. The wild type version of NRT1.13 does not transport nitrate in oocytes, when its S487P version does. Its mutation in plants triggers nitrate related phenotypes reminiscent to a transceptor activity. According to its localization and transcriptomic studies of the mutant, authors propose that NRT1.13 is able to monitor in planta NO^- concentrations and control nitrate allocation and flowering via a FLC-dependent pathway. This work is well performed and observations are of great interest for the Nitrate/Nitrogen community. The only remark I have is that it is difficult to draw a molecular mechanism by which NRT1.13 is involved in the sensing of NO^- and how NRT1.13 influences Nitrate allocation/flowering.

I would suggest answering this question by the following experiments. These are suggestions. Performing all the experiments is not mandatory for me to support the publication of this work. But having some hints concerning the molecular mechanism is needed, to my opinion.

It might be interesting to see if the S487P version if able to complement the flowering phenotypes. This will give us an idea of Nitrate transport contribution to the phenotype.

Response: Our oocyte-based studies in Figure 1 indicate that NRT1.13 does not function as a nitrate transporter, so nitrate transport activity does not contribute to the observed phenotype.

Is CHL1 WT and P492L versions under NRT1.13 promoter able to complement the phenotype? Same idea as above.

Response: Our preliminary data shows that CHL1 and NRT1.13 interact with different kinase family members, so they operate differently as a sensor. Therefore, it is likely that CHL1 cannot complement *nrt1.13*, as they probably elicit different responses.

Is Primary Nitrate Response (PNR) impacted in *nrt1.13* mutant? It would be nice to perform QPCR on PNR reporters in the conditions of Figure 5. This will give insights into the role of *nrt1.13* in controlling a well-defined NO^- related signaling pathway.

Response: As shown in Supplementary Figure 5, the primary nitrate response in the *nrt1.13* mutant is not affected.

The growth condition used for PNR is different from the condition applied for experiments in Figure 5. For the PNR assay, the plants have to be grown in media solely containing ammonia as nitrogen source under sterile conditions. However, to phenotype flowering (Figure 5), plants were grown in a hydroponic system.

Is pCHL1:NRT1.13 (WT and S487P) is able to complement chl1 mutants for PNR?

Response: As mentioned above, our preliminary data shows that CHL1 and NRT1.13 interact with different kinase family members, so they operate differently as a sensor. Therefore, they are not interchangeable.

Is NRT1.13 is really not a Nitrate transporter? Please provide a range of concentrations for Nitrate uptake in oocytes, since the partial complementation of the phenotype in planta could be explained by a partial nitrate transport activity not seen in oocytes.

Response: We have examined the high and low affinity nitrate uptake activity of NRT1.13 and found that NRT1.13 exhibits no nitrate transport activity under either conditions. To address the problem of partial complementation, we have isolated another NRT1.13 mutant, *nrt1.13-2*, and it displays a similar flowering phenotype (Supplementary Figure 4).

NRT1.13/NPF4.4 has already been shown as not being able to transport NO^- (Leran et al Scientific Report), but belongs to a sub-family with GA and ABA transporters. GAs are known flowering controllers. Thus, as discussed by the authors, it would be nice to test GA transport by NRT1.13...

Response: In 2015, Y. Chiba et al., (J plant Res 128, 679) reported the GA transport activity of the NRT1 family. Their results (Figure 2 of that study) show that NRT1.13 is not a GA transporter.

Is NRT1.13 a sort of "natural dominant negative" that will impact the activity of another NRT1/NPF? Can you co-inject CHL1 and NRT1.13 for instance and test nitrate transport? Or look for a NPF being co-expressed in the parenchyma cells and test interaction for a) nitrate transport [should explain the nitrate allocation phenotype] or b) hormone transport [could explain the flowering phenotype]...

Response: Our data shows that NRT1.13 is required for lateral nitrate transport at nodes, so it cannot be a dominant-negative partner.

Minor: p6, lines 115-117. Authors state that NRT1.13 is able to bind NO -. To my understanding of the work nothing support this conclusion. This should be rephrase or data supporting this conclusion should be provided.

Response: Acknowledged. We now present two lines of evidence to demonstrate that NRT1.13 can bind nitrate. The first is to show the nitrate transport activity of NRT1.13-S487P. Since residue S487 does not lie in the substrate binding pocket, the fact that NRT1.13-S487P exhibits nitrate transport activity indicates that the substrate binding pocket of wild type NRT1.13 can recognize nitrate. A respective description about the position of S487P has been added in line 125 of the revised manuscript. Secondly, to further confirm that wild type NRT1.13 can bind nitrate, we performed a microscale thermophoresis binding assay using purified NRT1.13 protein. As shown in new Supplementary Figure 3, the resulting binding isotherms reveal that NRT1.13 can bind nitrate.

Finally and most importantly, to my opinion NRT1.13 could probably be an active NO - transporter in plants, in peculiar conditions not reflected in oocyte experiments. Indeed, recent report has shown that P492L version of CHL1 is able to transport NO - when oocytes are pre-loaded with NO - (Noguero et al, https://www.biorxiv.org/content/early/2018/02/26/244467). The underlying hypothesis is that CHL1-P492L perceives internal-NO - at higher concentrations (with lower affinity) to switch the transporter activity on. If NRT1.13 is a "natural version" of that kind, NO - concentration in plant cells should be enough to activate NRT1.13 NO - transport activity and this would explain the *nrt1.13* mutant phenotype... I think authors need to test this hypothesis that could clearly explain why *nrt1.13* have a

NO - related phenotypes in planta. This can be made by preforming the same experiments as Noguero et al or following point 1,2,4 above.

Response: To address this concern, we injected nitrate into oocytes to attain a final concentration of ~10 mM KNO3. Under this condition, NRT1.13 still shows no nitrate uptake activity compared with water-injected oocytes, indicating that internal nitrate does not impact the properties of NRT1.13 or the S487P mutation.

Again this work is of very good quality but we are just missing information/hypothesis about the actual mechanism that will explain the link between oocytes and in planta observations. Any substantial explanation(s) following my suggestions above (or any other insight) should be provided for publication in high impact journal.

Reviewer #3

In this manuscript Chen et al show that AtNRT1.13 does not exhibit nitrate transport activity but can act as a transceptor. This is likely due to the exchange of proline 487 with a serine. Deletion mutants for AtNRT1.13 exhibit a pronounced phenotype under nitrogen starvation: delayed flowering, an increased node number as well as retarded branch outgrowth and nitrate allocation to nodes. This is a careful study describing the phenotype, cellular and tissue localization as well as effects on flowering time genes under nitrogen starvation of AtNRT1.13. Nevertheless, there are a few points that should be clarified:

The authors claim that AtNRT1.13 bind nitrate. Although this seems likely this is just a hypothesis. The best would be if they could proof such a binding, but I am aware that this is not easy. However at least a much more careful formulation should be used.

Could it be that AtNRT1.13 interacts was a nitrate transporting NRT and by doing so modulates its transport activity. If this cannot be excluded the authors should discuss also this possibility.

Response: Acknowledged. We now present two lines of evidence to demonstrate that NRT1.13 can bind nitrate. The first is to show the nitrate transport activity of NRT1.13-S487P. Since residue S487 does not lie in the substrate binding pocket, the fact that NRT1.13-S487P exhibits nitrate transport activity indicates that the substrate binding

pocket of wild type NRT1.13 can recognize nitrate. A respective description about the position of S487P has been added in line 125 of the revised manuscript. Secondly, to further confirm that wild type NRT1.13 can bind nitrate, we performed a microscale thermophoresis binding assay using purified NRT1.13 protein. As shown in new Supplementary Figure 3, the resulting binding isotherms reveal that NRT1.13 can bind nitrate.

The difference in nitrate content in the mutant implies that the transport activity for nitrate is reduced. The authors mention this fact in one sentence in the discussion, but I would like to get a deeper discussion. Is no other NRT deregulated? Or could this altered transport activity be due to an interaction of AtNRT1.13 with other NRTs as mentioned above?

Response: We have two transcriptome datasets, one for the leaf and one for the node. In both datasets, there is either no or very slight (non-significant) change for all NRT1 and NRT2 genes. For example, *CHL1* expression is 1.34-fold that of wild type level in the node of the *nrt1.13* mutant, but exhibits no difference in the leaf. In addition, expression of *NPF8.4*, which does not transport nitrate (our preliminary result), is 50% that of wild type level. We acknowledge that changes in posttranscriptional activation of transport and expression in a few critical cells cannot be revealed by transcriptomic analyses. A respective discussion of the potential mechanisms has now been added in line 453 of the revised Discussion.

Our unpublished Y2H results show that some NRT1 (NPF) transporters can interact with other NRT1s. There are several possibilities for the defect of *nrt1.13* in lateral N^{15}-nitrate allocation, including 1) altered expression of some unknown transporter, 2) altered post-transcriptional regulation of some transporters, or 3) as suggested, indirect influence on other nitrate transporters by protein-protein interactions. These possibilities are summarized in line 450 of our Discussion: "Since our functional study showed that NRT1.13 cannot transport nitrate directly (Figure 1), the allocation defect may be due to an indirect effect of NRT1.13 on either expression or activation of some unknown transporters through post-transcriptional regulation or protein-protein interactions."

− For CLCs it has been shown that a similar P to Ser mutations shifts the substrate specificity from NO3- to Cl-. I strongly suggest to investigate whether AtNRT1.13 does not act (also) as a chloride transporter/channel. Loss of such an activity may also have an impact on nitrate partitioning. In case AtNRT1.13 can transport chloride, chloride contents should be measured.

Response: According to the structure of CLCec1, AtCLCa residue P160 that is responsible for substrate selectivity lies in the transmembrane region and, more specifically, within the transport channel. Based on the CHL1 crystal structure, NRT1.13 residue S487, which corresponds to P492 in the CHL1 structure, is located in the cytosolic loop and, consequently, not in the substrate binding pocket. Therefore, it is unlikely that substrate selectivity will be changed by Pro-to-Ser mutation in NRT1.13. A respective description about the position of residue P492 has now been added to the revised manuscript in line 112.

TPC2020-RA-00771D 1st Editorial decision – *Accept with minor revisions* **Oct. 26, 2020**

On the basis of the advice received, the board of reviewing editors may accept your manuscript for publication in The Plant Cell, contingent on revision based on the comments of our reviewers.

As you will see, the reviewers were overall positive about this new version of your manuscript. However, in a post-review discussion that included a third consultant, it was agreed that the proposal that NRT1.13 is a tranceptor needs additional support. It remains possible that NRT1.13 provides a low level of transport that is sufficient for the phenotypes tested, or that the S487P mutation improves protein folding or trafficking in oocytes rather than affecting transport directly. Testing the in vivo functions of NRT1.13 (S487P) and its ability to complement the nrt1.13 mutants would be needed to address this caveat. Ideally, you would add this experiment; if you choose not to, we felt that it would be sufficient to alter the title to reflect that NRT1.13 as tranceptor remains a working model, and to add alternative explanations for your data in the discussion.

An additional note from the editors: We are trying to make a concerted effort to change green/red comparisons to green/magenta to make our figures understandable to those with color vision deficiencies. We noticed that a small number of your figures utilize red/green contrasts, so as you prepare the final version of the figures, please check the figures for red/green color use. Magenta is a good substitute for red, even if the data are labeled as "RFP" or

"mCherry" - readers will understand that colors can be changed, and indeed, it can be adjusted relatively quickly by using the "hue" setting in Photoshop or similar software. For example, the images may need to be corrected in Figure 2. Please note that color changes do not need to be highlighted or tracked in the revised manuscript, but could be noted in the cover letter or response document.).

-- Reviewer comments:

Reviewer #1 (Comments for the Author):

Thanks for great improvement of this manuscript by adding several new evidences. In particular, authors provided another T-DNA insertional mutant of NRT (nrt1.13-2) to support the NRT1.13 role in controlling flowering, used single flc and flc nrt1.13 double mutants to further strengthen NRT1.13-FLC regulatory pathway of flowering and branching, the new experiment to confirm the binding capacity of NRT1.13.

I don't understand why the authors did not directly respond my previous major concerns "It is very interesting to know the phenotype of complementation of nrt1.13 by expressing mutated NRT1.13 with S487P which might have dual functions in nitrate transport and sensing. Will it show complete recovery of the mutant and loss of the nitrate-independent manner? Since mutated NRT1.13 (S487P) could restore nitrate uptake, would it also rescue the flowering defect? How does NRT1.13 (S487P) respond to altered nitrate conditions?"

In view of that fact, I agree their major finding that AtNRT1.13 modulates shoot architecture and flowering time in a nitrate-dependent manner; however, I keep my reservation on concluding that AtNRT1.13 is a transceptor. My suggestion is either changing the title (remove 'transceptor') and toning down the conclusion/discussion or providing the complementation testing of NRT1.13 (S487P).

Moreover, it is not mandatory but may strengthen the finding by showing the data of partial complementation, not fully complementation of the transgenic line, in transcriptional level or posttranscriptional level; showing the nitrate levels in BS1 and CL1 (partially?) in addition to the flowering time restored by the complementation.

Minor comments:

1. In Figure 1. NRT1.13 shows no nitrate uptake activity, but S487P conversion 646 restores its nitrate transport activity. However, the error bar is quite small and it is questionable to conclude no significant difference of 15-nitrate between the oocytes expressing NRT1.13 and control at 5 mM nitrate supply. Please check original data and the statistics.

2. Lines 301-302: nitrate concentration exhibited little or no effect on node number of the primary 301 inflorescence stem, flowering time and basal branch outgrowth in Col-0. In fact, both Figure 4D and Figure S4C shows large difference between low and normal N for WT flowering time. The statistical difference could be significant if making the comparison only for WT between the two N regimes.

Reviewer #3 (Comments for the Author):

The authors invested a lot of time and energy to address the concerns of the referees. They did a careful work and from my side the ms can be accepted now.

TPC2020-RA-00771DR1 1st Revision received Nov. 30, 2020

Reviewer comments on previous submission and **author responses**:

We thank you for your time and effort in handling our manuscript.

We have modified this manuscript by:

1) Changing the title to "Potential transceptor AtNRT1.13 modulate shoot architecture and flowering time in a nitrate dependent manner;

2) Substituting red color by magenta color in Figure 2 to make the figure

understandable to those with color vision deficiencies;

3) Presenting STD instead of SE in Figure 1 to avoid confusion;

We have received reviews of your manuscript entitled "Transceptor AtNRT1.13 modulates shoot architecture and flowering time in a nitrate-dependent manner." On the basis of the advice received, the board of reviewing editors may accept your manuscript for publication in The Plant Cell, contingent on revision based on the comments of our reviewers.

As you will see, the reviewers were overall positive about this new version of your manuscript. However, in a post-review discussion that included a third consultant, it was agreed that the proposal that NRT1.13 is a tranceptor needs additional support. It remains possible that NRT1.13 provides a low level of transport that is sufficient for the phenotypes tested, or that the S487P mutation improves protein folding or trafficking in oocytes rather than affecting transport directly. Testing the in vivo functions of NRT1.13 (S487P) and its ability to complement the nrt1.13 mutants would be needed to address this caveat. Ideally, you would add this experiment; if you choose not to, we felt that it would be sufficient to alter the title to reflect that NRT1.13 as tranceptor remains a working model, and to add alternative explanations for your data in the discussion.

Response: Reviewer #1 said "It is very interesting to know the phenotype of complementation of nrt1.13 by expressing mutated NRT1.13 with S487P which might have dual functions in nitrate transport and sensing." It is something interesting to know but it cannot provide further evidence to demonstrate that NRT1.13 is a transceptor or not. If the flowering phenotype merely relies on the sensing function of NRT1.13 (transceptor model), NRT1.13 S487P with dual function will behave like NRT1.13 in flowering regulation. If the flowering phenotype relies on the tiny (if any) transport activity of NRT1.13 (transporter model), NRT1.13 S487P will behave better than NRT1.13 in flowering regulation. For either case, we will observe the recovery of the flowering phenotype in both NRT1.13 and NRT1.13 S487P complement lines, and the possibilities of being a transceptor or transporter cannot be distinguished. That is why we didn't perform this experiment, which will take at least one year, and get no additional information.

When you ectopic express a gene, there is always an issue of gene expression levels and protein expression levels. Under this condition, to ask a quantitative question of if NRT1.13 S487P behaves better than NRT1.13 or not in the flowering control, we need to make sure that both proteins are expressed at the same levels in the same critical cells. The expression levels need to be the same not only in the whole seedling but also more importantly in the few critical cells to make a fair comparison.

This will be a tough and almost impossible mission. In oocyte functional assay, we checked the protein expression level in a single cell to compare the transport activities of NRT1.13 and NRT1.13 S487P. And, we use GFP fusion to show that both proteins are properly targeted to the plasmamembrane. Therefore the concerns about transport activity and protein folding have been addressed.

We try our best to provide evidences needed. Two out of the three reviewers are satisfied with our revision, and we already address most of the questions of the reviewer #1 except this one. As I explain above, I don't think that the experiment of introducing NRT1.13 S487P into nrt1.13 mutant will provide additional information. I really hope that we can keep the original title. Nevertheless, if you think that without this experiment, the title needs to be changed, the title can be changed to "Potential transceptor AtNRT1.13 modulates shoot architecture and flowering time in a nitrate-dependent manner."

An additional note from the editors: We are trying to make a concerted effort to change green/red comparisons to green/magenta to make our figures understandable to those with color vision deficiencies. We noticed that a small number of your figures utilize red/green contrasts, so as you prepare the final version of the figures, please check the figures for red/green color use. Magenta is a good substitute for red, even if the data are labeled as "RFP" or "mCherry" - readers will understand that colors can be changed, and indeed, it can be adjusted relatively quickly by using the "hue" setting in Photoshop or similar software. For example, the images may need to be corrected in Figure 2. Please note that color changes do not need to be highlighted or tracked in the revised manuscript, but could be noted in the cover letter or response document.

Response: As suggested, red color is substituted by magenta color in Figure 2.

Reviewer #1 (Comments for the Author):

Thanks for great improvement of this manuscript by adding several new evidences. In particular, authors provided another T-DNA insertional mutant of NRT (nrt1.13-2) to support the NRT1.13 role in controlling flowering, used single flc and flc nrt1.13 double mutants to further strengthen NRT1.13-FLC regulatory pathway of flowering and branching, the new experiment to confirm the binding capacity of NRT1.13.

I don't understand why the authors did not directly respond my previous major concerns "It is very interesting to know the phenotype of complementation of nrt1.13 by expressing mutated NRT1.13 with S487P which might have dual functions in nitrate transport and sensing. Will it show complete recovery of the mutant and loss of the nitrate-independent manner? Since mutated NRT1.13 (S487P) could restore nitrate uptake, would it also rescue the flowering defect? How does NRT1.13 (S487P) respond to altered nitrate conditions?"

In view of that fact, I agree their major finding that AtNRT1.13 modulates shoot architecture and flowering time in a nitrate-dependent manner; however, I keep my reservation on concluding that AtNRT1.13 is a transceptor. My suggestion is either changing the title (remove 'transceptor') and toning down the conclusion/discussion or providing the complementation testing of NRT1.13 (S487P).

Response: As you pointed out earlier that "It is very interesting to know the phenotype of complementation of nrt1.13 by expressing mutated NRT1.13 with S487P which might have dual functions in nitrate transport and sensing." It is something interesting to know but it cannot provide further evidence to demonstrate that NRT1.13 is a transceptor or not. If the flowering phenotype merely relies on the sensing function of NRT1.13 (transceptor model), NRT1.13 S487P with dual function will behave like NRT1.13 in flowering regulation. If the flowering phenotype relies on the tiny (if any) transport activity of NRT1.13 (transporter model), NRT1.13 S487P will behave better than NRT1.13 in flowering regulation. For either case, we will observe the recovery of the flowering phenotype in both NRT1.13 and NRT1.13 S487P complement lines, and the possibilities of being a transceptor or transporter cannot be distinguished. That is why we didn't perform this experiment, which will take at least one year, and get no additional information.

When you ectopic express a gene, there is always an issue of gene expression levels and protein expression levels. Under this condition, to ask a quantitative question of if NRT1.13 S487P behaves better than NRT1.13 or not in the flowering control, we need to make sure that both proteins are expressed at the same levels in the same

critical cells. The expression levels need to be the same not only in the whole seedling but also more importantly in the few critical cells to make a fair comparison. This will be a tough and almost impossible mission. In oocyte functional assay, we checked the protein expression level in a single cell to compare the transport activities of NRT1.13 and NRT1.13 S487P. And, we use GFP fusion to show that both proteins are properly targeted to the plasmamembrane. Therefore the concerns about transport activity and protein folding have been addressed. The title is changed to "Potential transceptor AtNRT1.13 modulates shoot architecture and flowering time in a nitrate-dependent manner."

Moreover, it is not mandatory but may strengthen the finding by showing the data of partial complementation, not fully complementation of the transgenic line, in transcriptional level or posttranscriptional level; showing the nitrate levels in BS1 and CL1 (partially?) in addition to the flowering time restored by the complementation.

Minor comments:

1. In Figure 1. NRT1.13 shows no nitrate uptake activity, but S487P conversion restores its nitrate transport activity. However, the error bar is quite small and it is questionable to conclude no significant difference of 15-nitrate between the oocytes expressing NRT1.13 and control at 5 mM nitrate supply. Please check original data and the statistics.

Response: The error bar presented in the original Figure 1 is SE. To avoid the confusion, we change it to STD.

2. Lines 301-302: nitrate concentration exhibited little or no effect on node number of the primary inflorescence stem, flowering time and basal branch outgrowth in Col-0. In fact, both Figure 4D and Figure S4C shows large difference

between low and normal N for WT flowering time. The statistical difference could be significant if making the comparison only for WT between the two N regimes.

Response: In this sentence, we just want to emphasize that the effect of nitrate concentration on the flowering is more significant in the mutant. ANOVA analysis

showed that WT 2 mM and 0.2 mM nitrate are in the same statistic groups for flowering time (Figure, 4D, 4E), and node number (Figure 7A). The nitrate-dependent delay of basal branch outgrowth comparing between 2 mM and 0.2 mM nitrate in wild type is about 8 days, which is much lower than the delay of 23 days in the mutant. Similarly, the nitrate-dependent delay of flowering comparing between 2 mM and 0.2 mM nitrate in wild type is about 5 days, which is much lower than the delay of 12 days in the second mutant in Figure S4C. The sentence was modified as "nitrate concentration exhibited minor or no effect on node number of the primary inflorescence stem, flowering time and basal branch outgrowth in Col-0".

Reviewer #3 (Comments for the Author):

The authors invested a lot of time and energy to address the concerns of the referees. They did a careful work and from my side the ms can be accepted now.

Response: Thanks.

**TPC2020-RA-00771DR1 2nd Editorial decision – *revision requested***	**Dec. 7, 2020**

Thank you for submitting your response to the most recent reviews and the editor's evaluation.

In our last decision letter, we mentioned that in post-review discussions, reviewers and editors agreed that the data presented fall short of providing conclusive evidence that NRT1.13 is a transceptor. (Please note that at TPC, editorial decisions are not made strictly on the written reviews, but after post-review conversations with the reviewers and editors; in this case, we also pulled in an additional consultant). We asked you to either 1) provide additional evidence that NRT1.13 is a transceptor, or 2) "alter the title to reflect that NRT1.13 as tranceptor remains a working model, and to add alternative explanations for your data in the discussion." While you did provide an new title, no changes were made in the rest of document to tone down these claims.

We would like to give you another chance to revise the manuscript and avoid over-interpretation and to thoughtfully present alternative hypotheses throughout the paper.).

**TPC2020-RA-00771DR2 2nd Revision received**	**Jan. 1, 2021**

Reviewer comments on previous submission and **author responses**:

According to your suggestions, we have modified this manuscript by:

1) Adding the statement: "Thus, these data suggest that NRT1.13 may function as a transceptor to monitor nitrate levels in the xylem and regulate the plasticity of shoot architecture. Nevertheless, we cannot completely exclude the alternative possibility that NRT1.13 without transport activity might interact with other nitrate transporters in planta to modulate nitrate distribution, leading to the observed developmental changes." in lines 329-334, marked in blue.

2) Adding the statement: "Single amino acid substitutions in a protein may alter protein stability or targeting. Nevertheless, when we injected the same amount of cRNA into Xenopus oocytes, the protein expression levels of wild type NRT1.13 and S487P were similar (Supplementary Figure 2), indicating that it is more likely that substitution of S487 with proline does not affect protein stability. In addition, both wild type NRT1.13 and S487P-GPF localize in the plasma membrane upon transient expression in mesophyll protoplasts (Figure 2). Therefore, the lack of nitrate transport activity of wild type NRT1.13 is not due to changes in protein stability or targeting and, instead, it is more likely that wild-type NRT1.13 is a defective nitrate transporter. Thus, unless an as yet unknown partner protein, present only in planta but not in Xenopus oocytes, is required to restore the conformation and transport activity of NRT1.13, the available data indicates that transport activity might not be required for the function of NRT1.13 to regulate shoot development and flowering plasticity." in lines 480-493, marked in blue. 3) Adding the localization study of NRT1.13 S487P in Figure 2. In our previous revision, we already present alternative explanations as:

(A) “Since our functional study showed that NRT1.13 cannot transport nitrate directly (Figure 1), the allocation defect may be due to an indirect effect of NRT1.13 on either expression or activation of some unknown transporters through post-transcriptional regulation or protein-protein interactions.” in line 455 – 458, highlighted in yellow.

(B) “Although NRT1.13 has shown no ABA, GA or JA-Ile transport ability (Kanno et al., 2012; Chiba et al., 2015) and the proline residue in CHL1 is important for auxin transport (Krouk et al., 2010), the possibility for NRT1.13 without the corresponding proline residue to transport hormones might be low but cannot be completely ruled out.” In line 473 – 477.

TPC2020-RA-00771DR2 3rd Editorial decision – *acceptance pending* **Jan. 9, 2021**

We are pleased to inform you that your paper entitled "Potential transceptor AtNRT1.13 modulates shoot architecture and flowering time in a nitrate-dependent manner" has been accepted for publication in The Plant Cell, pending a final minor editorial review by journal staff. At this stage, your manuscript will be evaluated by a Science Editor with respect to its presentation of scientific content, compliance with journal policies, and presentation for a broad readership.

Final acceptance from Science Editor **Feb. 3, 2021**
